# Atrial Fibrillation in Retinal Artery Occlusion: A Systematic Review and Meta-Analysis

**DOI:** 10.3390/medsci14050553

**Published:** 2026-09-08

**Authors:** Aurelia Mihaela Nica, Madalina Andreea Beldie, Samer Andrei Nica, Minela Aida Maranduca, Cosmin Victor Ganea, Paula Cristina Morariu, Maria Mihaela Godun, Roxana Elena Ciuntu, Camelia Margareta Bogdanici, Mariana Floria

**Affiliations:** 1 Grigore T. Popa University of Medicine and Pharmacy Iasi, 16 University Street, 700115 Iasi, Romania; nica_aurelia-mihaela@d.umfiasi.ro (A.M.N.); madalina-andreea.beldie@umfiasi.ro (M.A.B.); samer-andrei.nica@d.umfiasi.ro (S.A.N.); cosmin-victor.ganea@umfiasi.ro (C.V.G.); morariu.paula-cristina@email.umfiasi.ro (P.C.M.); godun.maria-mihaela@d.umfiasi.ro (M.M.G.); camelia.bogdanici@umfiasi.ro (C.M.B.); floria.mariana@umfiasi.ro (M.F.); 2 Ophthalmology, 2nd Surgery Department, Saint Spiridon Emergency Hospital, 700111 Iasi, Romania; 3 Centre for Translational Medicine, Semmelweis University, 1085 Budapest, Hungary; 4 Internal Medicine Clinic, Saint Spiridon Emergency Hospital, 700111 Iasi, Romania

**Keywords:** atrial fibrillation, retinal artery occlusion, central retinal artery occlusion, electrocardiography, cardiac monitoring, prevalence, meta-analysis, systematic review

## Abstract

**Background/Objectives**: Retinal artery occlusion (RAO) represents an ischemic event of the anterior circulation, driven by impaired blood flow through the ophthalmic branches of the internal carotid artery. Atrial fibrillation (AF) is an increasingly recognized cardioembolic source in patients with RAO, yet its prevalence remains poorly characterized. We conducted a systematic review and meta-analysis to estimate the pooled prevalence of AF in RAO patients and to summarize evidence on its incidence and association with this condition. **Methods**: Four databases were searched through April 2025, updated on 18 August 2026. Studies reporting AF prevalence, incidence, or association in adults with RAO were included. Pooled prevalence was estimated using the Freeman–Tukey double arcsine transformation with a random-effects model. Subgroup analyses, meta-regression, and sensitivity analyses were performed to explore heterogeneity. **Results**: Sixty-two studies were included in the qualitative synthesis, of which 58 reported AF prevalence data. Among the 36 studies comprising 139,222 patients, the pooled AF prevalence was 11.53% (95% CI: 9.41–13.82; I^2^ = 99.2%; prediction interval: 2.00–26.9%). AF prevalence was significantly higher in patients aged ≥70 years (15.86%) and in central RAO cohorts (16.54%). Incident AF following RAO ranged from 10.5% to 41%, increasing progressively with monitoring intensity. The pooled odds ratio for the AF–RAO association was 1.56 (95% CI: 1.06–2.29). **Conclusions**: AF affects approximately one in nine patients with RAO, though with substantial uncertainty across populations (prediction interval 2.00–26.9%); the extent to which this exceeds the general-population estimates independent of age, cardiovascular risk profile, and geographic factors remains uncertain. These findings support cardiac rhythm surveillance following RAO diagnosis in selected patients, although its effect on clinical outcomes remains to be established prospectively.

## 1. Introduction

Retinal artery occlusion (RAO) represents an anterior circulation ischemic stroke resulting from reduced perfusion within the ophthalmic arterial branches of the internal carotid system, leading to acute, painless, and often irreversible monocular vision loss [1,2]. RAO includes two main clinical entities: central retinal artery occlusion (CRAO) and branch retinal artery occlusion (BRAO) [1,2].

Most non-arteritic RAO cases are embolic in origin and share similar pathophysiological mechanisms with ischemic stroke [3,4,5]. Accordingly, the American Heart Association/American Stroke Association (AHA/ASA) classified acute retinal ischemia as a form of central nervous system infarction in their 2013 scientific statement, establishing the concept of RAO as a stroke-equivalent condition [6]. Clear recommendations for stroke-oriented diagnostic evaluation emerged later with the American Academy of Ophthalmology Preferred Practice Pattern for Retinal and Ophthalmic Artery Occlusions (AAO PPP) published in 2019 [7], the 2021 AHA/ASA scientific statement on CRAO [8], and the 2024 update of the AAO PPP [1], which emphasize urgent systemic assessment, including vascular imaging and cardiac evaluation, to identify potential embolic sources and guide secondary prevention.

Embolic sources in RAO have traditionally been attributed to carotid atherosclerotic disease [9,10,11]. However, cardiac sources are increasingly recognized as clinically relevant contributors, reflecting a broader systemic vascular process rather than a single-site pathology [2,12,13]. Among these, atrial fibrillation (AF) represents the most common sustained cardiac arrhythmia with embolic potential [14,15]. Given that AF is frequently asymptomatic and may remain undetected in the absence of systematic cardiac evaluation, its prevalence in patients with RAO may be substantially underestimated [16,17]. Identifying AF in this context is clinically important, as appropriate anticoagulation significantly reduces the risk of recurrent thromboembolic events [2,16,18,19].

Several studies have reported that AF is more prevalent among patients with RAO than in the general population [20,21]. Two systematic reviews and meta-analyses have previously addressed this relationship. Kewcharoen et al. published the first such analysis in 2019, reporting a pooled AF prevalence of 12.5% in CRAO patients and evaluating the association between AF and retinal vessel occlusion across both arterial and venous subtypes [20]. More recently, Chen et al. examined the association between AF and retinal vascular occlusions, reporting an odds ratio of 1.74 for RAO patients relative to the controls [22]. However, neither analysis focused exclusively on RAO, and since their publication, the evidence base has expanded considerably with numerous new observational studies reporting data on AF in this population. Reported prevalence estimates remain highly variable across studies, limiting the consistency and clinical interpretability of the existing evidence.

Therefore, we conducted a systematic review and meta-analysis to estimate the pooled prevalence of AF among adults presenting with RAO, and to summarize additional evidence on its incidence following RAO and its association with this condition.

## 2. Materials and Methods

### 2.1. Protocol and Registration

This systematic review and meta-analysis was conducted in accordance with the Preferred Reporting Items for Systematic Reviews and Meta-Analyses (PRISMA) 2020 guidelines [23]. The completed checklist is presented in Table S7. The study protocol was registered in the International Prospective Register of Systematic Reviews (PROSPERO) under the registration number CRD420250647949. Minor deviations from the registered protocol were made and are described in the corresponding sections of the Methods.

### 2.2. Literature Search Strategy

Two authors (A.M.N. and M.A.B.) independently performed a comprehensive literature search in the PubMed (MEDLINE), Embase, Scopus, and Web of Science databases from inception to 5 April 2025. The search was subsequently updated on 18 August 2026 to capture additional eligible studies published in the interim. The search strategy combined terms related to RAO and AF. The full search strategies for each database are provided in Tables S1–S4. Reference lists of the included studies and relevant reviews were also screened to identify additional eligible articles.

### 2.3. Eligibility Criteria

We included observational and interventional studies enrolling adult patients (≥18 years) with a confirmed diagnosis of RAO, including CRAO and BRAO, that reported the prevalence or incidence of AF by any detection method. Studies that additionally included a comparator group of individuals without RAO contributed to the analysis of the association between AF and RAO. Specific inclusion criteria were then applied separately to each of the three components of the review: the prevalence or incidence of AF in RAO, and the association between AF and RAO.

Studies were included in the AF prevalence component if they:

- were cohort or cross-sectional studies;
- reported the number or the proportion of patients with AF present at the time of the index RAO event, with a denominator comprising the entire RAO cohort;
- ascertained AF within the predefined window applied to the primary outcome (Section 2.4).

Studies were included in the incident AF after RAO component if they:

- were longitudinal studies with follow-up after the index RAO event;
- enrolled patients with RAO and no AF at baseline, with pre-existing AF excluded from the denominator;
- reported the number or the proportion of patients with AF newly diagnosed during a defined follow-up period;
- reported the method and duration of cardiac rhythm monitoring and the length of follow-up.

Studies were included in the association between AF and RAO component if they:

- were case–control or cohort studies including a comparator group of individuals without RAO, as defined by the original study;
- reported either an adjusted effect estimate with its 95% confidence interval, or sufficient raw data to derive a crude odds ratio.

A single study could contribute to more than one component provided the corresponding criteria were met; overlapping populations were never included within the same quantitative synthesis.

Studies were excluded if they:

1. did not clearly differentiate RAO from other retinal vascular diseases;
2. were case reports or case series with fewer than 10 patients;
3. were animal studies;
4. were conference abstracts or non–peer-reviewed publications;
5. were systematic reviews or meta-analyses (although their reference lists were screened to identify additional eligible studies).

No restrictions were applied regarding publication year, language, study setting or geographic location.

### 2.4. Outcome Definition

The primary outcome was the prevalence of AF among patients with RAO, defined as AF present at the time of the RAO event. The allowable ascertainment window for this outcome extended from any time before the index event to the completion of the initial diagnostic evaluation, defined as the index hospitalization or an equivalent protocolized work-up initiated at the time of the index event, with monitoring duration among contributing studies extending up to approximately 30 days. Prevalent AF therefore comprised AF diagnosed at any time before the index event together with AF first documented within this window. Newly detected AF cases identified through selective, heterogeneous screening initiated after the initial evaluation had concluded were not included in the primary pooled estimate. Case series and case–control studies, whose sampling frames preclude valid prevalence estimation, and cohort studies whose denominator did not represent the source RAO population, were not eligible for this component; these were retained in the qualitative synthesis.

Two secondary outcomes were assessed. The first was the incidence of AF after RAO, defined as newly diagnosed AF occurring during follow-up among patients without AF at baseline. The second was the association between AF and RAO, defined as the odds of AF in patients with RAO relative to individuals without RAO. Both secondary outcomes were additional to the registered protocol. These were analyzed where sufficient data were available but were not pre-specified and are therefore considered exploratory.

### 2.5. Study Selection & Screening Process

Records retrieved from the database searches were exported to EndNote for duplicate removal and subsequently uploaded to Rayyan (Qatar Computing Research Institute) for title and abstract screening. Two reviewers (A.M.N. and M.A.B.) independently and blindly screened titles and abstracts, followed by full-text assessment of potentially eligible studies. Disagreements were resolved through discussion, or when necessary, by consultation with the senior reviewers (M.F. and C.M.B.). Studies published in languages other than English were translated using DeepL to enable appropriate screening and evaluation.

### 2.6. Data Extraction

Six authors (A.M.N., M.A.B., S.A.N., M.A.M., C.V.G, and R.E.C.) were grouped into three pairs and independently extracted data using a standardized data collection form in Microsoft Excel. The following variables were collected:

- Study characteristics: first author, year of publication, country, study design, study setting, and timeframe of data collection;
- Population and disease characteristics: type of RAO, sample size, mean or median age, and proportion of male participants;
- Atrial fibrillation data: detection method and reported prevalence or incidence.

When studies reported the proportion or percentage of patients with AF rather than the absolute number of cases, the number of AF cases was calculated by multiplying the reported proportion or percentage by the total number of RAO participants.

When multiple publications reported data from the same cohort, the most recent study with the largest sample size was included to maximize data completeness and avoid duplication. Two publications originated from overlapping nationwide registry cohorts. In such cases, the cohort study was used for prevalence estimation, whereas the case–control study was included exclusively in the association analysis. Overlapping populations were not included within the same quantitative synthesis.

### 2.7. Risk-of-Bias Assessment

Two reviewers (P.C.M. and M.M.G.) independently assessed risk-of-bias using the Joanna Briggs Institute (JBI) critical appraisal tools according to study design, including the JBI checklists for prevalence studies (applied to cohort and cross-sectional designs), case series, and case–control studies. Overall risk-of-bias ratings were reached by consensus, applying a consistent principle across all JBI tools whereby domains bearing on whether a study was methodologically fit for its intended purpose carried greater weight than the remaining domains, with disagreements resolved through discussion. Although the specific items differ across the JBI checklists applied for the different study designs, this principle was applied uniformly and is consistent with JBI guidance. Detailed per-study risk-of-bias assessments are presented in Figures S1–S3.

### 2.8. Statistical Analysis

All statistical analyses were conducted using R software (version 4.5.2; R Foundation for Statistical Computing, Vienna, Austria) with the meta package (version 8.2-1).

The prevalence of AF in RAO was estimated using an inverse-variance weighted random-effects meta-analysis of proportions. Variance was stabilized using the Freeman–Tukey double arcsine transformation to account for studies with extreme prevalence estimates [24].

Between-study heterogeneity was assessed using Cochran’s Q (χ^2^) test and quantified using the I^2^ statistic, with *p* values < 0.05 considered indicative of statistically significant heterogeneity [25]. I^2^ values of 25%, 50%, and 75% were interpreted as low, moderate, and high heterogeneity, respectively [26]. A random-effects model with restricted maximum likelihood estimation was applied throughout, given the expected clinical and methodological diversity among studies.

Pooled estimates were back-transformed and presented as proportions with corresponding 95% confidence intervals. Prediction intervals were calculated to reflect the expected range of true prevalence in comparable settings.

Pre-specified subgroup analyses were performed to explore potential sources of heterogeneity, including geographic region, type of RAO, method of diagnosis of AF, age group, study design, study setting, timing of data collection, number of centers, sample size, and risk of bias. Differences between subgroups were assessed using the Q test for heterogeneity across subgroups.

Meta-regression analyses were conducted to further evaluate the influence of study-level characteristics on the variability of prevalence estimates. The proportion of between-study variance explained by each covariate was quantified using the coefficient of determination (R^2^).

Sensitivity analyses were performed using a leave-one-out approach to assess the robustness of the pooled estimate. In addition, a Baujat plot was used to identify studies contributing most to heterogeneity and influence on the overall effect.

Funnel plot asymmetry was visually assessed as an indicator of potential small-study effects.

Egger’s regression test was performed to evaluate publication bias, with *p* values < 0.1 considered indicative of statistically significant asymmetry [27].

For the association analysis, pooled odds ratios (ORs) and corresponding 95% confidence intervals (CIs) were calculated using an inverse-variance weighted random-effects model with the restricted maximum likelihood (REML) estimator, based on log-transformed effect estimates.

## 3. Results

### 3.1. Study Selection

Initially, we retrieved 3440 records from four electronic databases, including PubMed (n = 851), Scopus (n = 493), Embase (n = 1900), and Web of Science (n = 196). After the removal of 1042 duplicates, 2398 records were screened based on title and abstract and 2264 were excluded as not meeting the eligibility criteria. A total of 134 full-text articles were selected for detailed evaluation; 3 could not be retrieved, leaving 131 assessed for eligibility. Of these, 71 were excluded for specific reasons: conference abstracts (n = 33), non-original studies (n = 8), non-RAO populations (n = 11), wrong outcomes (n = 9), RAO data not reported separately (n = 4), case series including fewer than 10 patients (n = 4) and overlapping datasets (n = 2). An additional 10 records identified through citation screening were assessed, of which 2 met the inclusion criteria and were added to the final dataset.

**Figure 1:**
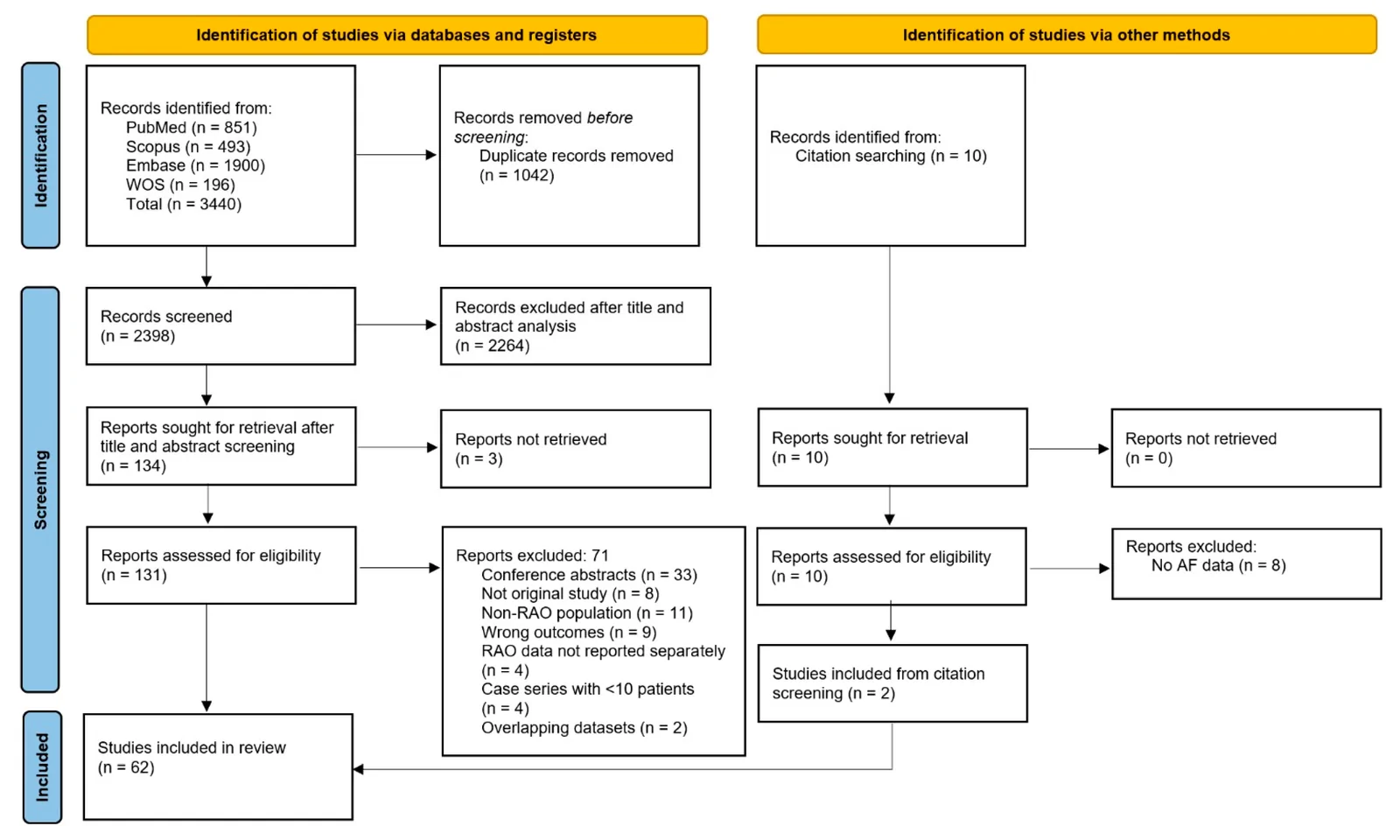
PRISMA 2020 flowchart of the selection process. AF = atrial fibrillation; RAO = retinal artery occlusion; WOS = Web of Science.

Ultimately, 62 studies met the inclusion criteria and were included in the systematic review, comprising 60 studies identified through database searches and 2 through citation screening. The study selection process is illustrated in Figure 1.

### 3.2. Study Characteristics

Of the 62 included studies, 58 reported data on AF prevalence [21,28,29,30,31,32,33,34,35,36,37,38,39,40,41,42,43,44,45,46,47,48,49,50,51,52,53,54,55,56,57,58,59,60,61,62,63,64,65,66,67,68,69,70,71,72,73,74,75,76,77,78,79,80,81,82,83,84], of which 1 also reported data on AF incidence [84]. An additional 4 studies reported exclusively on AF incidence following RAO [17,85,86,87]. Study characteristics are summarized in Table 1, with detailed study-level characteristics provided in Tables S5 and S6.

Most studies were published from 2020 onwards (n = 43, 69.4%) and were conducted in Europe (n = 30, 48.4%) and North America (n = 23, 37.1%). Cohort studies were the most common design (n = 26, 41.9%), followed by cross-sectional studies (n = 16, 25.8%), and most studies were hospital-based (n = 40, 64.5%).

AF detection methods varied, with most studies relying on medical records (n = 27, 43.5%) or administrative coding (n = 20, 32.3%), while fewer used extended cardiac monitoring (n = 8, 12.9%) or ECG alone (n = 7, 11.3%). Most studies included mixed RAO populations (n = 37, 59.7%), and the majority were assessed as having low (n = 27, 43.5%) or moderate (n = 26, 41.9%) risk of bias.

### 3.3. Pooled Prevalence of Atrial Fibrillation in Retinal Artery Occlusion

Across the 58 studies reporting AF prevalence, estimates varied widely, ranging from 0% to 28.6%. Of these, 36 cohort and cross-sectional studies comprising 139,222 patients provided estimates suitable for quantitative synthesis. The remaining studies were retained in the qualitative synthesis but not pooled: case series and case–control studies whose designs preclude valid prevalence estimation, and three studies were excluded because they did not yield a representative, uniformly ascertained estimate of AF prevalence. In the first of these, Kang et al., the source population had been systematically depleted of patients on recent antiplatelet therapy, a group enriched for AF, which artificially lowered the observed prevalence; AF was moreover captured as a pre-existing comorbidity coded in the year preceding the event rather than ascertained at the time of the occlusion [42]. In the second, Alhayek et al., the denominator comprised only the small subset of patients selected for thrombolysis (20 out of 563) rather than a representative RAO cohort [75]. Third, in Ryan et al., trial eligibility criteria excluded patients receiving effective oral or parenteral anticoagulation, the standard treatment for known AF, thereby underrepresenting patients with AF in the eligible population [82].

**Figure 2:**
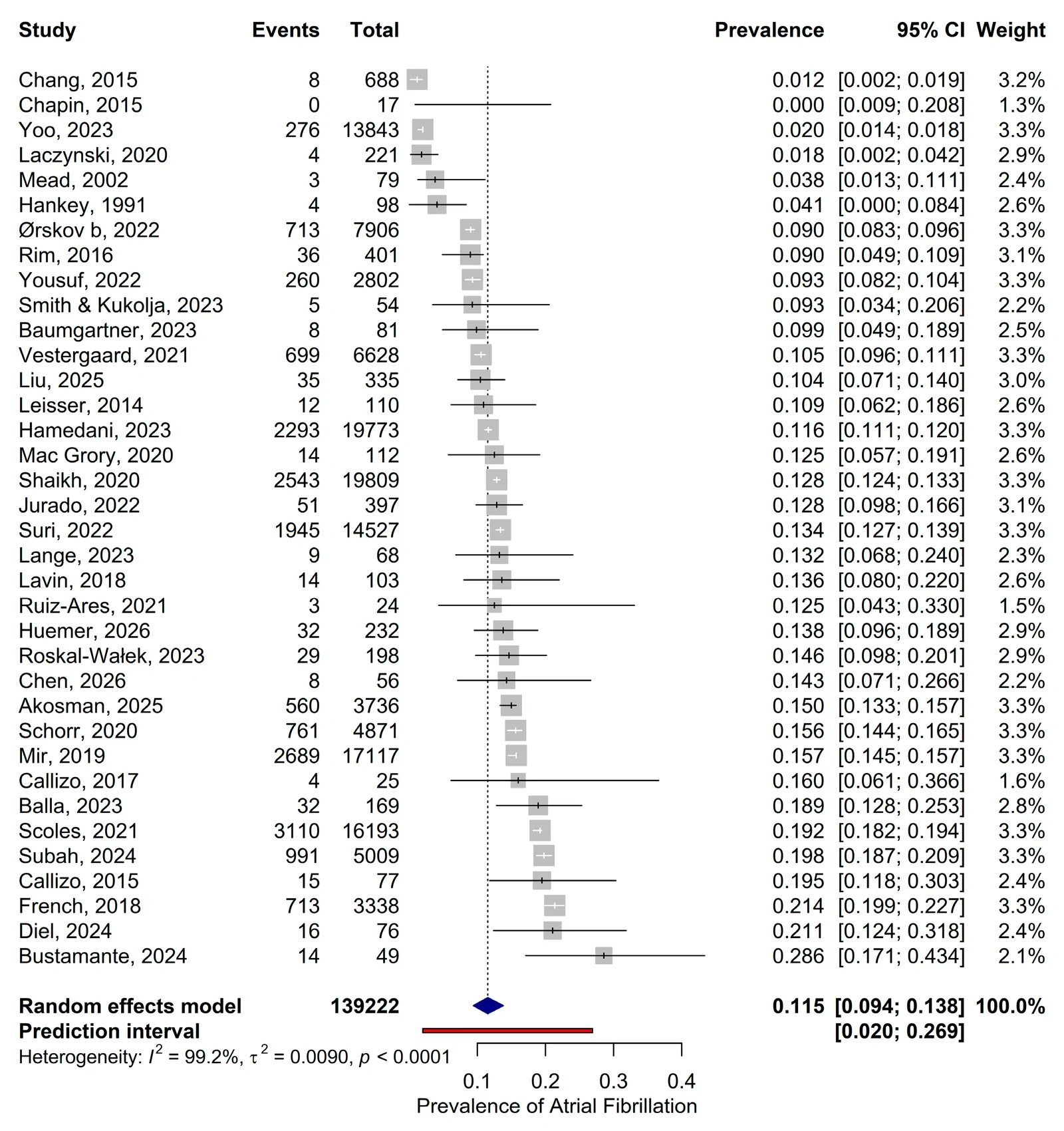
Forest plot showing the pooled prevalence of atrial fibrillation in retinal artery occlusion across the 36 included studies [19,21,28,29,30,34,36,37,38,39,41,44,45,46,47,48,50,53,55,56,57,62,64,66,68,69,71,72,73,74,76,77,78,81,83,84].

The pooled prevalence was 11.53% (95% CI: 9.41–13.82), with considerable between-study heterogeneity (I^2^ = 99.2%, *p* < 0.0001), as shown in Figure 2.

As a sensitivity analysis, the pooled prevalence was re-estimated using a generalized linear mixed model (binomial-normal random-effects model, logit link), an approach considered more robust than the Freeman–Tukey double arcsine transformation when sample sizes vary substantially across studies [88]. The GLMM yielded a pooled prevalence of 10.88% (95% CI: 8.72–13.51%; I^2^ = 98.5%), closely consistent with the primary estimate of 11.53% (95% CI: 9.41–13.82%), supporting the robustness of the pooled estimate to the choice of pooling method (Figure S15).

Subgroup analyses demonstrated significant variation across several study characteristics (Table 2 and Figures S4–S14).

Prevalence differed by geographic region (*p* = 0.034), with lower estimates observed in Asia-Pacific populations compared to Europe and North America (Table 2; Figure S4).

When stratified by type of RAO, prevalence was significantly higher in studies including only CRAO compared to mixed RAO populations (*p* < 0.0001) (Table 2; Figure S5).

Age was also a significant determinant, with higher estimates in populations aged 70 years and over than in younger populations (*p* = 0.019) (Table 2; Figure S6).

Prevalence varied according to method of diagnosis of AF, with higher estimates observed in studies using extended cardiac monitoring compared to electrocardiography, medical records, or administrative coding; however, subgroup differences were not statistically significant (*p* = 0.400) (Table 2; Figure S7).

Prevalence additionally differed by study period (*p* = 0.041), with studies whose enrollment midpoint fell in 2013 or later yielding higher estimates than earlier studies (13.61% vs. 8.96%) (Table 2; Figure S8).

Additional subgroup analyses by study setting, timing of data collection, number of centers, risk of bias, study design, and sample size did not reveal statistically significant differences (all *p* > 0.05) and are presented in Table 2 and Figures S9–S14.

Meta-regression analysis, performed separately for each moderator, identified age, geographic region, and type of RAO as significant contributors to between-study heterogeneity (age: R^2^ = 39.3%, *p* < 0.0001; geographic region: R^2^ = 29.0%, *p* = 0.002; RAO type: R^2^ = 20.6%, *p* = 0.003) (Table 3).

**Figure 3:**
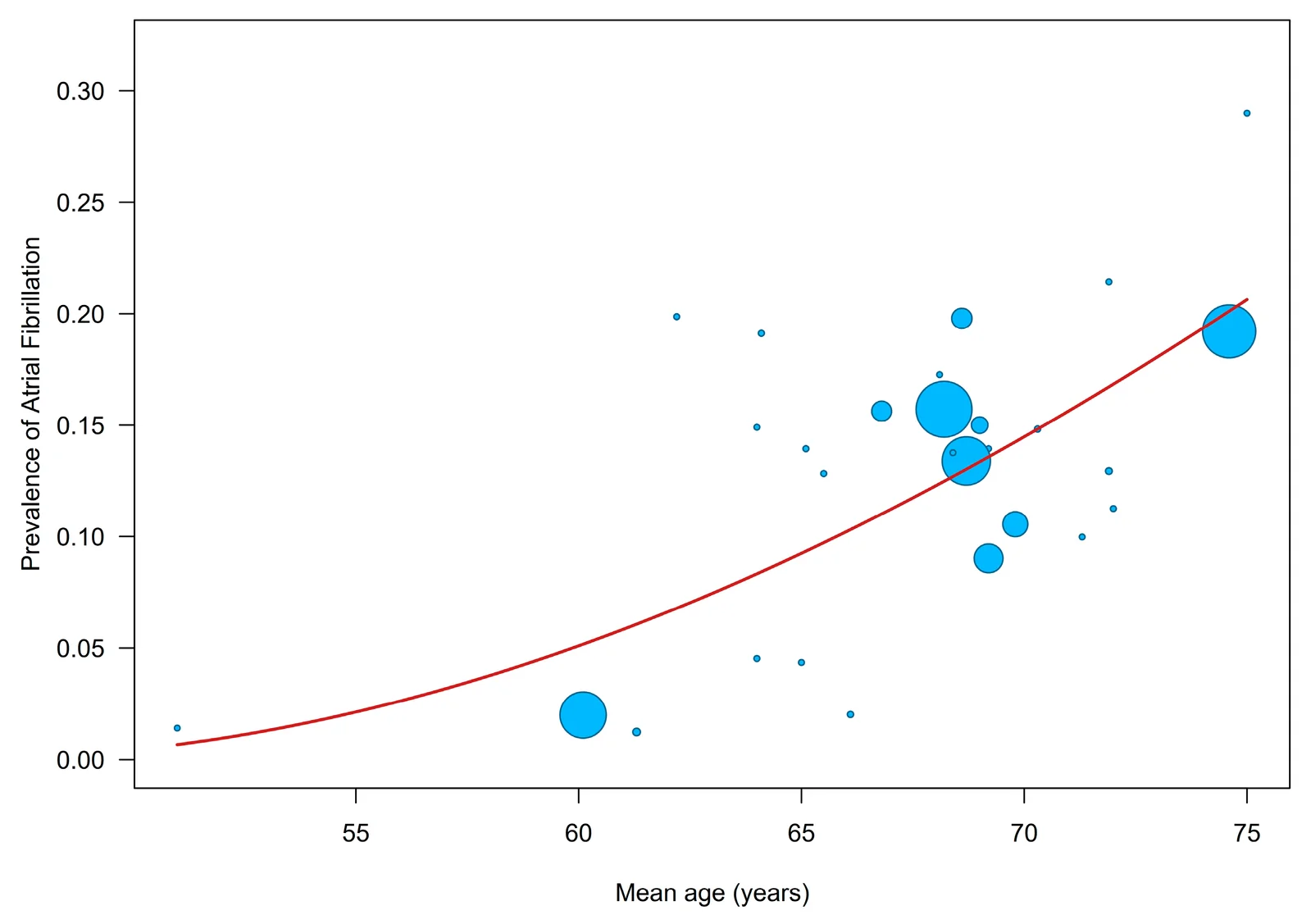
Bubble plot of the association between mean age and prevalence of atrial fibrillation showing increasing atrial fibrillation prevalence with older mean study age (R^2^ = 39.3%).

The relationship between age and AF prevalence is illustrated in Figure 3 while corresponding estimates for geographic region and RAO type are presented as bubble plots in Figures S16 and S17.

A multivariable model combining all three moderators explained 59.7% of the heterogeneity (k = 28) (Table 3; Figure S18), with each moderator remaining independently significant (*p* < 0.05). As these are study-level associations, they should be interpreted as ecological rather than individual-level effects. Method of AF diagnosis and other study-level variables did not significantly explain heterogeneity (all R^2^ = 0%).

Sensitivity analysis using a leave-one-out approach showed that no individual study had a disproportionate influence on the pooled estimate (Figure S19), as further illustrated by the Baujat plot (Figure S21).

Visual inspection of the funnel plot did not reveal clear asymmetry (Figure S20), and Egger’s test did not demonstrate statistically significant small-study effects (*p* = 0.92). However, given the extreme between-study heterogeneity (I^2^ = 99.2%), this result should not be interpreted as strong evidence against publication bias.

### 3.4. Incidence of AF After RAO

Five studies evaluated the incidence of newly diagnosed AF following RAO, including 1258 patients (Table 4; Table S6) [17,84,85,86,87].

Four studies included mixed RAO cohorts [17,84,86,87], while one study focused exclusively on CRAO [85]. All studies were retrospective cohort studies conducted in Europe or the United States, with sample sizes ranging from 46 to 706 patients.

Methods of AF detection varied across studies, ranging from administrative coding [17,84] and standard electrocardiography or 24-h Holter monitoring [86] to implantable loop recorders [87] and prolonged implantable cardiac monitoring [85], with incident AF detected in 10.5%, 11.2%, 12.6%, 15.2%, and 41% of patients, respectively, suggesting a progressive increase in detection rates with more intensive and prolonged cardiac monitoring strategies. Due to the substantial methodological and clinical heterogeneity across studies, a quantitative synthesis was not performed.

### 3.5. Association Between Atrial Fibrillation and Retinal Artery Occlusion

**Figure 4:**
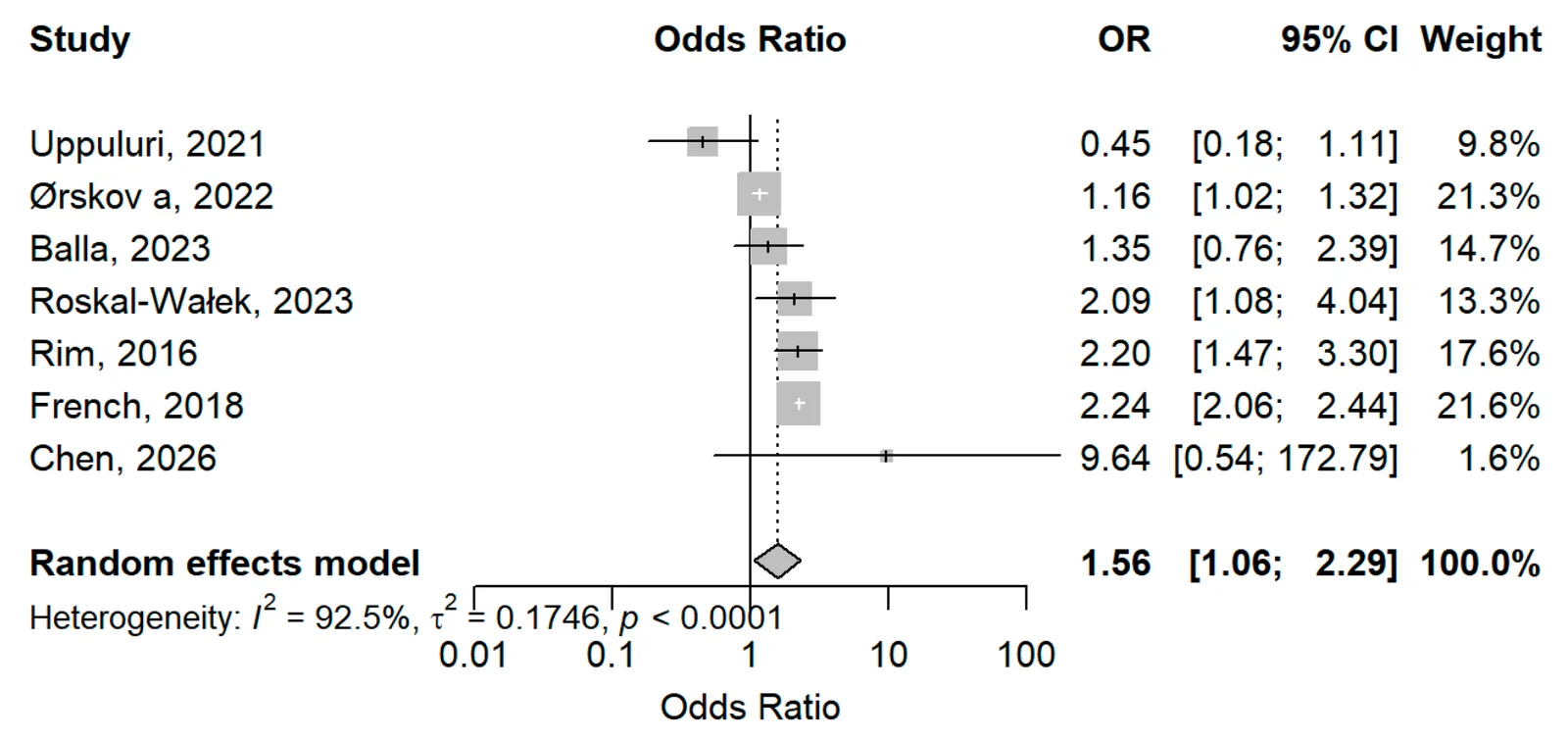
Forest plot of the association between atrial fibrillation and retinal artery occlusion [39,41,51,59,66,69,83].

The association analysis included seven studies, with 9996 patients with RAO and 26,309,908 individuals in the non-RAO control groups [39,41,51,59,66,69,83]. The pooled analysis showed higher odds of AF in patients with RAO compared to the controls (OR = 1.56, 95% CI 1.06–2.29) (Figure 4).

Substantial heterogeneity was observed (I^2^ = 92.5%, τ^2^ = 0.1746, *p* < 0.0001). Leave-one-out sensitivity analysis yielded pooled estimates ranging from OR = 1.41 to OR = 1.76, with point estimates remaining directionally consistent across all iterations, although statistical significance was not retained when four out of the seven studies were excluded individually (Figure S22). Restricting the analysis to the six studies reporting unadjusted (crude) estimates yielded a pooled OR of 1.68 (95% CI 1.06–2.66, I^2^ = 68.3%), similar in magnitude to the primary estimate (Figure S23).

## 4. Discussion

### 4.1. Principal Findings

This systematic review and meta-analysis included 62 studies in the qualitative synthesis. Of these, 58 reported AF prevalence data, of which 36 studies comprising 139,222 patients were eligible for quantitative synthesis, yielding a pooled AF prevalence of 11.53% (95% CI: 9.41–13.82; I^2^ = 99.2%) in patients with RAO, corresponding to approximately one in nine individuals, with a prediction interval of 2.00% to 26.9% and substantial uncertainty across settings and populations. This extreme between-study heterogeneity underscores that the pooled estimate should be interpreted as a central tendency across highly diverse populations rather than a precise, generalizable figure.

These findings build upon and substantially extend the prior evidence base. Kewcharoen et al. published the first meta-analysis on this topic in 2019, reporting a pooled AF prevalence of 12.5% (95% CI: 4.7–20.3) in CRAO patients [20]. More recently, Chen et al. evaluated the occurrence of AF across retinal vascular occlusions, encompassing both arterial and venous subtypes, without deriving a pooled prevalence estimate specific to RAO [22]. Our prevalence estimate is broadly consistent with these earlier reports. While both prior analyses addressed retinal artery and retinal vein occlusion as part of a shared scope, neither focused exclusively on RAO, a condition with a predominantly embolic substrate that is pathophysiologically distinct from retinal vein occlusion and more directly linked to cardioembolism. The present analysis extends this evidence base by focusing exclusively on RAO, incorporating a larger and more contemporaneous body of literature, and integrating findings from prevalence, incidence, and association analyses to provide a more comprehensive characterization of the relationship between AF and RAO.

### 4.2. Atrial Fibrillation Prevalence in Retinal Artery Occlusion Versus the General Population

The present analysis identified a pooled AF prevalence of 11.53% among patients with RAO, substantially exceeding the estimates reported in the general population. In a large, geographically and ethnically diverse United States cohort, Alonso et al. reported an overall AF prevalence of 5.9% [89], while epidemiological data indicate that incident cases of AF are doubling every few decades [90]. Globally, incident AF cases peak at 65–69 years and prevalent cases at 75–79 years, reflecting a strong age-dependent pattern [91]. This elevated AF burden is, in part, consistent with the known demographic profile of RAO patients, as RAO itself predominantly affects older individuals, with mean age at diagnosis of 63.6 years across all subtypes [92] and 74.3 years in CRAO cohorts [93]. Nevertheless, at nearly twice the general population estimate, the present findings suggest that the concentration of AF in RAO patients exceeds what would be anticipated on the basis of age alone, pointing to a clinically relevant association between the two conditions. However, this comparison should be interpreted with caution: RAO cohorts in the present analysis were substantially older on average, drawn from heterogeneous geographic regions and healthcare settings, and enriched for cardiovascular risk factors relative to general-population samples. The present data do not establish that the elevated prevalence observed in RAO patients is attributable to RAO itself, independent of these demographic and clinical differences.

### 4.3. Incident Atrial Fibrillation Following Retinal Artery Occlusion

Across studies reporting incident AF following RAO, the proportion of patients diagnosed with AF increased with the intensity and duration of cardiac monitoring, ranging from approximately 10% to 41%. The yield of cardiac monitoring therefore depends on both its duration and the patient selected. Brief monitoring windows miss most cases, as both studies using implantable monitoring detected only a small fraction of eventual AF within the first 30 days, with the majority of diagnoses emerging later [85,87]. Real-world workup nonetheless remains limited, as Vonderlin et al. found that only half of RAO patients underwent any cardiological evaluation and that roughly one third of those with documented AF were not anticoagulated according to the guidelines [86], a treatment gap also observed in a broader cohort of ischemic monocular visual loss [94]. Watson et al. further identified older age, a higher CHA2DS2-VASc score, prolonged PR interval, and mitral annular calcification as predictors of subsequent AF, indicating that extended monitoring offers the greatest yield in patients with a higher cardiovascular risk profile [87]. A further study using administrative ICD-10 coding reported a comparable incident AF rate of 11.2% within the subgroup of patients with a hospital admission during follow-up [84]. Unlike the other studies, incident cases were not distinguished through active cardiac monitoring but through routinely collected hospital diagnostic codes, and prevalent AF was not excluded prior to ascertainment, limiting direct comparability with the monitoring-based estimates above.

Across all five studies, monitoring strategy, patient population, and follow-up duration differed substantially; the apparent relationship between monitoring intensity and AF detection should not be interpreted as causal, nor does this analysis address clinical outcomes. These findings support evaluating RAO with the same diagnostic urgency applied to ischemic stroke, with the consideration of prolonged rhythm monitoring in selected higher-risk patients.

### 4.4. Association Between AF and RAO

Our association analysis demonstrated higher odds of AF among patients with RAO compared with non-RAO controls (OR = 1.56, 95% CI 1.06–2.29), consistent in direction with previously reported odds ratios of 2.24 and 1.74 [20,22]. This directional concordance across independent datasets is robust; the apparent magnitude of the association, however, is highly sensitive to the extent of adjustment for shared vascular risk factors.

A nationwide Taiwanese study reported a substantially higher adjusted hazard ratio of 8.32 (95% CI 3.70–18.32) but accounted only for age and sex [95]. In contrast, when Lusk et al. progressively adjusted a large administrative cohort for demographic and then vascular comorbidities, an initially positive crude hazard ratio of 2.55 attenuated and ultimately reversed to 0.72 (95% CI 0.60–0.87), a reversal the authors attributed to incomplete ascertainment of RAO in outpatient settings [96]. A subsequent analysis by the same group, incorporating outpatient claims and propensity-score overlap weighting across more than one million beneficiaries, identified a small but independent association (adjusted HR 1.14, 95% CI 1.02–1.28) while cautioning that residual confounding could not be excluded [97].

Taken together, these findings indicate that a substantial component of the crude association is attributable to the shared vascular risk profile of the two conditions. Whether AF constitutes an independent risk factor for RAO, rather than merely a marker of shared vascular burden, cannot be determined from the available evidence, as only one of the seven included studies provided an adjusted estimate. Notably, this single adjusted study reported a more modest association (OR 1.16, 95% CI 1.02–1.33) than the pooled crude estimate derived from the remaining six studies (OR 1.68, 95% CI 1.06–2.66), a pattern consistent with attenuation upon adjustment. This interpretation reinforces the case for a comprehensive vascular evaluation following RAO rather than one narrowly focused on the detection of AF alone.

### 4.5. Adequacy of Cardiac Workup

Despite increasing adherence to guideline-recommended evaluation, stroke workup in RAO remains suboptimal [71], with more than half of the patients not undergoing any form of brain, carotid, or cardiac assessment [56,81,98,99]. Previous reports have shown that cardiac testing was performed in only 23.8% of cases [56], while subsequent studies have reported higher rates of 40.28% [81] and 50.6% [86]. In a recent study, echocardiography was obtained in 12.50% of patients, whereas electrocardiography was performed in 27.78% of cases, with ECG utilization increasing from 21.47% in 2016 to 31.60% in 2021 [81]. Taken together, these findings suggest that the true prevalence of AF in patients with RAO is likely underestimated, given both the suboptimal rates of cardiac evaluation and the inherent limitations of standard detection strategies in identifying paroxysmal arrhythmia. In this context, the RAO event may serve not only as a consequence of pre-existing AF, but also as a sentinel manifestation prompting its detection, particularly in patients with paroxysmal or previously undiagnosed arrhythmia.

### 4.6. Sources of Heterogeneity

The substantial between-study heterogeneity observed in the primary analysis (I^2^ = 99.2%) reflects the clinical and methodological diversity inherent to the included studies. Meta-regression analyses, performed separately for each moderator, identified mean study age, geographic region, and RAO subtype as significant contributors to between-study variance, and combined in a multivariable model, these three moderators jointly accounted for 59.7% of the observed heterogeneity (Table 3, Figures S16–S18). As these represent study-level associations, they should be interpreted as ecological rather than individual-level effects and cannot be assumed to reflect the relationship between age, region, or RAO subtype and AF risk within individual patients.

Age was the strongest determinant of AF prevalence across studies (R^2^ = 39.3%, *p* < 0.0001; Figure 3). Subgroup analysis confirmed this pattern, with studies enrolling predominantly older populations (mean age ≥ 70 years) yielding a pooled AF prevalence of 15.86% (95% CI: 12.30–19.76) compared with 9.95% (95% CI: 7.02–13.31) in studies enrolling younger populations (mean age < 70 years, *p* = 0.019). This nearly twofold difference is consistent with the well-established age-dependent trajectory of AF and underscores the importance of accounting for study population age when interpreting prevalence estimates across studies [90]. The same gradient is apparent at the lower extreme of the age spectrum: in a case–control study confined to young adults aged 20–45 years, AF or atrial flutter was present in only 1.0% of patients with RAO and did not exceed its frequency among non-occlusion controls (odds ratio 0.49, 95% CI: 0.21–1.16) [51].

Geographic region was the second most important contributor to heterogeneity (R^2^ = 29.0%, *p* = 0.002). Prevalence estimates were markedly lower in Asia-Pacific studies (4.71%, 95% CI: 1.15–10.44) compared with European (12.38%, 95% CI: 9.82–15.18) and North American cohorts (13.12%, 95% CI: 10.31–16.19), a pattern consistent with the lower overall AF burden documented in Asian populations in global epidemiological studies [91]. Differences in cardiovascular risk factor profiles, genetic predisposition, and diagnostic practices across regions may partly account for this geographic variation.

RAO subtype was the third significant contributor (R^2^ = 20.6%, *p* = 0.003). Studies restricted to CRAO reported a pooled AF prevalence of 16.54%, nearly double that observed in mixed RAO cohorts (9.64%). This finding likely reflects a dilution effect, as CRAO is more strongly associated with cardioembolic mechanisms compared with BRAO, which more commonly arises from local atherosclerotic or small vessel disease, thereby attenuating pooled AF prevalence estimates in mixed cohorts. Beyond the cardioembolic pathway, evidence on the relationship between AF and retinal microvascular changes remains heterogeneous. Earlier studies did not identify an independent association between retinal microvascular alterations and AF [100,101,102], whereas more recent data have demonstrated impaired retinal microvascular endothelial function even in patients in sinus rhythm [19,103], pointing toward a more complex systemic vascular substrate underlying the AF–AO relationship.

Study period was also a significant contributor: studies whose enrollment midpoint fell in 2013 or later reported a substantially higher pooled AF prevalence than earlier studies (13.61% vs. 8.96%, *p* = 0.041). This threshold coincides with the AHA/ASA statement classifying acute retinal ischemia as a stroke-equivalent condition [6], and the observed shift more plausibly reflects the progressive adoption of systematic post-RAO cardiovascular evaluation than a true change in the underlying prevalence of AF.

The method of AF detection did not significantly explain between-study variance in meta-regression (R^2^ = 0%); however, subgroup analysis revealed a clinically meaningful gradient, with studies employing extended cardiac monitoring reporting higher AF prevalence (16.43%) than those relying on standard ECG (8.59%). This finding likely reflects the well-recognized phenomenon of paroxysmal AF underdetection with brief or single-timepoint monitoring strategies [15], and has direct implications for the design of cardiac evaluation protocols following RAO.

### 4.7. Clinical Implications

These findings have direct implications for the clinical management of patients with RAO. Ensuring adherence to a systematic, stroke-like evaluation pathway should be a priority, as current evidence suggests a gap between guideline recommendations and real-world practice. Beyond this, given the substantial cardiovascular and cerebrovascular risk burden in this population, greater emphasis should be placed on structured screening for atrial fibrillation as part of the cardiac workup. Current guidelines recommend routine heart rhythm assessment during healthcare contact in individuals aged ≥ 65 years, and prolonged non-invasive ECG screening in those aged ≥75 years or aged ≥ 65 years with additional CHA_2_DS_2_-VA risk factors [15]; given the older age profile of patients with RAO, the majority already meet these thresholds and would warrant AF screening on age grounds alone, irrespective of the index occlusive event. Implementation of clear, standardized care pathways and closer collaboration between ophthalmology, neurology, and cardiology may further improve the consistency and quality of care. Whether improved AF detection in this population translates into reduced thromboembolic events has not been directly evaluated by this review and remains to be established prospectively.

### 4.8. Strengths and Limitations

This study has several important strengths. It represents the largest synthesis to date of evidence on AF specifically in RAO, with a large number of included studies and patients allowing for more precise estimates and improved generalizability of the findings. The use of subgroup and meta-regression analyses enabled a more detailed exploration of between-study heterogeneity, identifying key determinants such as age, geographic region, and RAO subtype. Finally, by integrating evidence on incident AF and its association with RAO, our study offers a broader perspective on the role of AF across the clinical spectrum of this condition.

Several limitations should be acknowledged. The included studies were heterogeneous in design, population characteristics, and methods of AF detection, and substantial residual heterogeneity remained despite systematic exploration of its sources. In many studies, AF ascertainment relied on administrative coding or chart review without detailed information on diagnostic methods, which may have affected accuracy and comparability, and potentially led to the underestimation of true AF prevalence. Planned subgroup analyses based on cardiovascular comorbidities were not feasible due to inconsistent definitions and reporting across studies. Similarly, data on antiplatelet and anticoagulant therapy, smoking status, and prior thromboembolic history at the time of the index RAO event were inconsistently reported and could not be systematically extracted, precluding assessment of the adequacy of pre-event medical management. Finally, for the association analysis, crude data were used in several studies due to the limited availability of adjusted estimates, which may have restricted the ability to account for potential confounding. These limitations highlight the need for prospective studies employing standardized cardiac monitoring protocols in well-characterized RAO populations.

## 5. Conclusions

This systematic review and meta-analysis demonstrates that AF affects approximately one in nine patients with RAO, with a pooled prevalence of 11.53% (I^2^ = 99.2%; prediction interval 2.00–26.9%). Prevalence varied markedly with patient age, RAO subtype, geographic region, and study period, and the extent to which it exceeds general-population estimates independent of these factors remains uncertain. The convergent evidence from prevalence, incidence, and association analyses supports the concept of RAO as a sentinel event for underlying AF, frequently undetected by conventional evaluation strategies. These findings support cardiac rhythm surveillance following RAO diagnosis in selected patients, although its effect on clinical outcomes remains to be established prospectively. Future studies should focus on standardizing cardiac monitoring protocols in RAO patients and evaluating their impact on clinical outcomes.

## Figures and Tables

**Table 1:** Summary of the characteristics of the 62 included studies.

Characteristics	N = 62
Publication year	
Range	1991–2026
1991–2019	19 (30.6%)
≥2020	43 (69.4%)
Geographic region	
Europe	30 (48.4%)
North America	23 (37.1%)
Asia-Pacific	9 (14.5%)
Study design	
Cohort	26 (41.9%)
Cross-sectional	16 (25.8%)
Case series	13 (21.0%)
Case–control	6 (9.7%)
RCT	1 (1.6%)
Study setting	
Hospital-based	40 (64.5%)
Registry-based	21 (33.9%)
Population-based	1 (1.6%)
Centers	
Multicenter	32 (51.6%)
Single-center	30 (48.4%)
Atrial fibrillation diagnostic procedure	
Medical records	27 (43.5%)
Administrative coding	20 (32.3%)
Extended cardiac monitoring	8 (12.9%)
ECG	7 (11.3%)
Type of RAO	
RAO	37 (59.7%)
CRAO	25 (40.3%)
Risk of bias	
Low	27 (43.5%)
Moderate	26 (41.9%)
High	9 (14.5%)

RCT = randomized controlled trial; ECG = electrocardiogram; CRAO = central retinal artery occlusion; RAO = retinal artery occlusion.

**Table 2:** Pooled prevalence of atrial fibrillation in retinal artery occlusion and subgroup analyses by geographic region, RAO subtype, AF diagnostic method, age group, study design, study setting, timing of the data collection, number of centers, risk of bias, study period, and sample size.

	Studies (n)	Participants (n)	Prevalence (%)	95% CI	Heterogeneity		P For Subgroup Differences
					I^2^	*p* Value	
Overall	36	139,222	11.53	9.41–13.82	99.2	<0.0001	-
Geographic region							0.034
Europe	17	16,271	12.38	9.82–15.18	77.3	<0.0001	
Asia-Pacific	4	15,267	4.71	1.15–10.44	96.7	<0.0001	
North America	15	107,684	13.12	10.31–16.19	98.3	<0.0001	
Type of RAO							<0.0001
CRAO	11	30,030	16.54	14.25–18.96	91.0	<0.0001	
Mixed RAO	25	109,192	9.64	7.32–12.24	99.3	<0.0001	
Atrial fibrillation detection method							0.400
Administrative coding	16	136,873	11.69	8.66–15.10	99.6	<0.0001	
Medical records	12	1782	10.99	7.61–14.86	80.9	<0.0001	
ECG	4	278	8.59	2.41–17.56	78.9	<0.0001	
Extended cardiac monitoring	4	289	16.43	10.08–23.87	51.5	<0.0001	
Age group *							0.019
<70 years	21	75,374	9.95	7.02–13.31	99.4	<0.0001	
≥70 years	7	17,077	15.86	12.30–19.76	76.7	<0.0001	
Study design							0.167
Cohort	20	65,232	10.62	7.61–14.05	99.5	<0.0001	
Cross-sectional	16	73,990	13.17	11.10–15.39	96.0	<0.0001	
Study setting							0.848
Hospital-based	20	2184	11.49	8.58–14.75	78.0	<0.0001	
Registry-based	16	137,038	11.64	8.63–15.03	99.6	<0.0001	
Timing of data collection							0.501
Retrospective	27	138,347	11.25	8.94–13.77	99.4	<0.0001	
Prospective	9	875	12.84	7.79–18.85	76.0	<0.0001	
Number of centers							0.851
Single-center	15	1829	11.63	8.11–15.64	82.0	<0.0001	
Multicenter	21	137,393	11.51	8.88–14.41	99.5	<0.0001	
Risk-of-bias							0.280
Low	15	132,984	10.85	7.65–14.53	99.7	<0.0001	
Moderate	13	5343	11.85	8.26–15.94	84.8	<0.0001	
High	8	895	13.88	11.19–16.79	44.9	<0.0001	
Study period **							0.041
Before 2013	16	72,369	8.96	6.03–12.37	98.5	<0.0001	
2013 and later	18	66,696	13.61	10.82–16.65	99.4	<0.0001	
Sample size							0.331
Below 200	17	1396	12.53	9.54–15.84	63.0	<0.0001	
200 and larger	19	137,826	11.03	8.27–14.15	99.6	<0.0001	

* Eight studies were excluded from the age subgroup analysis due to missing data. CI = confidence interval; ** Stratified by study-period midpoint (mean of enrollment start and end years), dichotomized at 2013; two studies were excluded from this subgroup analysis due to missing enrolment date data; CRAO = central retinal artery occlusion; RAO = retinal artery occlusion; ECG = electrocardiogram.

**Table 3:** Meta-regression of age, geographic region, and type of retinal artery occlusion as predictors of atrial fibrillation prevalence.

	k	Coefficient	95% CI	*p*	R^2^
Univariable models					
Age (per year)	28	0.0163	0.0081 to 0.0244	<0.0001	39.3%
Region—Europe vs. Asia-Pacific	36	0.1490	0.0563 to 0.2416	0.002	29.0%
Region—North America vs. Asia-Pacific	36	0.1549	0.0632 to 0.2467	<0.001	
RAO type—Mixed RAO vs. CRAO	36	−0.1010	−0.1679 to −0.0342	0.003	20.6%
Multivariable model					
Age (per year)	28	0.0106	0.0021 to 0.019	0.014	59.7% *
Region—Europe vs. Asia-Pacific	28	0.1353	0.0093 to 0.2614	0.035	
Region—North America vs. Asia-Pacific	28	0.1434	0.0195 to 0.2672	0.023	
RAO type—Mixed RAO vs. CRAO	28	−0.0668	−0.1315 to −0.0021	0.043	

* R^2^ for the multivariable model reflects variance jointly explained by all three moderators combined. Coefficients on the Freeman–Tukey double arcsine transformed scale; k = number of studies (28 for models including age, due to 8 studies with missing age data).

**Table 4:** Characteristics of the studies reporting the incidence of atrial fibrillation following retinal artery occlusion.

Author	Year	Country	Study Design	Type of RAO	Sample Size	Incident AF	Method of Diagnosis of AF	AF Definition	Follow-Up Duration
Christiansen [17]	2018	Denmark	Retrospective cohort	RAO	706	74	ICD-8; ICD-10	NS	Until AF, death, or end of study
Watson [87]	2020	United States	Retrospective cohort	RAO	46	7	Implantable loop recorder	Episode of irregular rhythm without P waves detected by ILR (≥2 min)	43.2 months
Mac Grory [85]	2021	United States	Retrospective cohort	CRAO	100	41	Implantable cardiac monitoring (ICM, IPG, ICD, CRT-P, CRT-D)	AF episode ≥ 2 min detected by implantable cardiac device	24 months
Vonderlin [86]	2021	Germany	Retrospective cohort	RAO	174	22	ECG or ≥24-h Holter	AF documented by ECG or Holter	20 ± 12 months
Huemer [84]	2026	United Kingdom	Retrospective cohort	CRAO/ BRAO	136/96	19/7	ICD-10	NS	NS

RAO = retinal artery occlusion; AF = atrial fibrillation; ICD-8 = International Classification of Diseases, 8th revision; ICD-10 = International Classification of Diseases, 10th revision; NS = not specified; ILR = implantable loop recorder; CRAO = central retinal artery occlusion; EHR = electronic health record; ICM = insertable cardiac monitor; IPG = implantable pacemaker generator; ICD = implantable cardioverter-defibrillator; CRT-P = cardiac resynchronization therapy device-pacemaker; CRT-D = cardiac resynchronization therapy device-defibrillator.

## Data Availability

All data analyzed in this study were derived from previously published articles, which are cited in the manuscript. The extracted data supporting the findings are available within the article and its Supplementary Materials. Additional data are available from the corresponding author upon reasonable request.

## Supplementary Materials

The following supporting information can be downloaded at: https://www.mdpi.com/article/10.3390/medsci14050553/s1, Table S1: Search strategy for PubMed (MEDLINE); Table S2: Search strategy for Scopus; Table S3: Search strategy for Embase; Table S4: Search strategy for Web of Science; Table S5: Characteristics of studies reporting the prevalence of atrial fibrillation in retinal artery occlusion; Table S6: Characteristics of studies reporting incident atrial fibrillation after retinal artery occlusion; Table S7: PRISMA 2020 Checklist; Figure S1: Risk-of-bias traffic-light plot based on the Joanna Briggs Institute checklist for prevalence studies (cohort and cross-sectional designs); Figure S2: Risk-of-bias traffic-light plot based on the Joanna Briggs Institute checklist for case series; Figure S3: Risk-of-bias traffic-light plot based on the Joanna Briggs Institute checklist for case-control studies; Figure S4: Prevalence of atrial fibrillation in retinal artery occlusion by region; Figure S5: Prevalence of atrial fibrillation in retinal artery occlusion by type of retinal artery occlusion; Figure S6: Prevalence of atrial fibrillation in retinal artery occlusion by mean age of study participants; Figure S7: Prevalence of atrial fibrillation in retinal artery occlusion by method of diagnosis of atrial fibrillation; Figure S8: Prevalence of atrial fibrillation in retinal artery occlusion by study period; Figure S9: Prevalence of atrial fibrillation in retinal artery occlusion by study setting; Figure S10: Prevalence of atrial fibrillation in retinal artery occlusion by timing of data collection; Figure S11: Prevalence of atrial fibrillation in retinal artery occlusion by number of centers; Figure S12: Prevalence of atrial fibrillation in retinal artery occlusion by risk of bias in individual studies; Figure S13: Prevalence of atrial fibrillation in retinal artery occlusion by study design; Figure S14: Prevalence of atrial fibrillation in retinal artery occlusion by sample size; Figure S15: Sensitivity analysis re-estimating the pooled prevalence of atrial fibrillation using a generalised linear mixed model; Figure S16: Bubble plot of atrial fibrillation prevalence by type of retinal artery occlusion; Figure S17: Bubble plot of atrial fibrillation prevalence by geographic region; Figure S18: Forest plot of coefficients from the multivariable meta-regression model; Figure S19: Leave-one-out sensitivity analysis illustrating the influence of each individual study on the pooled prevalence estimate; Figure S20: Funnel plot evaluating potential publication bias among studies included in the meta-analysis of atrial fibrillation prevalence in retinal artery occlusion; Figure S21: Baujat plot showing the contribution of individual studies to heterogeneity in the prevalence meta-analysis; Figure S22. Leave-one-out sensitivity analysis of the pooled association between atrial fibrillation and retinal artery occlusion; Figure S23. Sensitivity analysis restricted to studies reporting crude estimates of the association between atrial fibrillation and retinal artery occlusion.

## Abbreviations

The following abbreviations are used in this manuscript:

AAO	American Academy of Ophthalmology
AF	Atrial fibrillation
AHA	American Heart Association
ASA	American Stroke Association
BRAO	branch retinal artery occlusion
CHA_2_DS_2_-VASc	Congestive heart failure, hypertension, age ≥ 75 years, diabetes mellitus, stroke/TIA, vascular disease, age 65–74 years, sex category (female)
CI	Confidence interval
CRAO	Central retinal artery occlusion
CRT-D	Cardiac resynchronization therapy device-defibrillator
CRT-P	Cardiac resynchronization therapy device-pacemaker
ECG	Electrocardiography
EHR	Electronic health record
ESC	European Society of Cardiology
GLMM	Generalized linear mixed model
I^2^	I-squared (heterogeneity statistic)
ICD	Implantable cardioverter-defibrillator
ICM	Insertable cardiac monitor
ILR	Implantable loop recorder
IPG	Implantable pacemaker generator
JBI	Joanna Briggs Institute
NS	Not specified
OR	Odds ratio
PPP	Preferred Practice Pattern
PRISMA	Preferred Reporting Items for Systematic Reviews and Meta-Analyses
PROSPERO	International Prospective Register of Systematic Reviews
R^2^	Coefficient of determination
REML	Restricted maximum likelihood
RAO	Retinal artery occlusion
