# Supplementary material for: Atrial Fibrillation in Retinal Artery Occlusion: A Systematic Review and Meta-Analysis
Source: Med Sci (Basel). 2026 Sep 8;14(5):553. doi: 10.3390/medsci14050553 (PMC13608936; doi:10.3390/medsci14050553)
Supplement: Supplementary file 1 [file medsci-14-00553-s001.zip › Supplementary Materials.pdf]

SUPPLIMENTARY MATERIAL

Contents

Table S1. Search strategy for PubMed (MEDLINE).
Table S2. Search strategy for Scopus.
Table S3. Search strategy for Embase.
Table S4. Search strategy for Web of Science.
Table S5. Characteristics of studies reporting the prevalence of atrial fibrillation in retinal artery occlusion.
Table S6. Characteristics of studies reporting incident atrial fibrillation after retinal artery occlusion.
Figure S1. Risk-of-bias traffic light plot based on the Joanna Briggs Institute checklist for prevalence studies (cohort and cross-sectional designs).

Figure S2. Risk-of-bias traffic light plot based on the Joanna Briggs Institute checklist for case series.

Figure S3. Risk-of-bias traffic light plot based on the Joanna Briggs Institute checklist for case–control studies.

Figure S4. Prevalence of atrial fibrillation in retinal artery occlusion by region.

Figure S5. Prevalence of atrial fibrillation in retinal artery occlusion by type of retinal artery occlusion.

Figure S6. Prevalence of atrial fibrillation in retinal artery occlusion by mean age of study participants.

Figure S7. Prevalence of atrial fibrillation in retinal artery occlusion by method of diagnosis of atrial fibrillation.

Figure S8. Prevalence of atrial fibrillation in retinal artery occlusion by study period.

Figure S9. Prevalence of atrial fibrillation in retinal artery occlusion by study setting.

Figure S10. Prevalence of atrial fibrillation in retinal artery occlusion by the timing of data collection.

Figure S11. Prevalence of atrial fibrillation in retinal artery occlusion by number of centers.

Figure S12. Prevalence of atrial fibrillation in retinal artery occlusion by risk of bias in individual studies.

Figure S13. Prevalence of atrial fibrillation in retinal artery occlusion by study design.

Figure S14. Prevalence of atrial fibrillation in retinal artery occlusion by sample size.

Figure S15: Sensitivity analysis re-estimating the pooled prevalence of atrial fibrillation using a generalised linear mixed model.

Figure S16. Bubble plot of atrial fibrillation prevalence by type of retinal artery occlusion.

Figure S17. Bubble plot of atrial fibrillation prevalence by geographic region.

Figure S18. Forest plot of coefficients from the multivariable meta-regression model.

Figure S19. Leave-one-out sensitivity analysis illustrating the influence of each individual study on the pooled prevalence estimate.

Figure S20. Funnel plot evaluating potential publication bias among studies included in the meta-analysis of atrial fibrillation prevalence in retinal artery occlusion.

Figure S21. Baujat plot showing the contribution of individual studies to heterogeneity in the prevalence meta-analysis.

Figure S22. Leave-one-out sensitivity analysis of the pooled association between atrial fibrillation and retinal artery occlusion.

Figure S23. Sensitivity analysis restricted to studies reporting crude estimates of the association between atrial fibrillation and retinal artery occlusion.

Table S1. Search strategy for PubMed (MEDLINE).
Original search: April 5, 2025, n = 770. Updated search: August 18, 2026, n = 851.

1.	(("Atrial" AND "Fibrillation") OR "AF" OR (cardiac AND arrhythmia) OR ("irregular" AND "heartbeat") OR (heart AND "rhythm disorder"))
2.	((Retinal AND (Artery AND (Occlusion OR embolism))) OR "RAO" OR "CRAO" OR "BRAO" OR ((Retinal OR Ocular) AND Stroke))
3.	1 and 2
4.	Limit to humans (applied via PubMed filter)

Table S2. Search strategy for Scopus.

Original search: April 5, 2025, n = 431. Updated search: August 18, 2026, n = 493.

1.	TITLE-ABS-KEY (("Atrial" AND "Fibrillation") OR "AF" OR (cardiac AND arrhythmia) OR ("irregular" AND "heartbeat") OR (heart AND "rhythm disorder"))
2.	TITLE-ABS-KEY ((Retinal AND (Artery AND (Occlusion OR embolism))) OR "RAO" OR "CRAO" OR "BRAO" OR ((Retinal OR Ocular) AND Stroke))
3.	1 and 2

Table S3. Search strategy for Embase.

Original search: April 5, 2025, n = 1,594. Updated search: August 18, 2026, n = 1,900.

1.	(("Atrial" AND "Fibrillation") OR "AF" OR (cardiac AND arrhythmia) OR ("irregular" AND "heartbeat") OR (heart AND "rhythm disorder"))
2.	((Retinal AND (Artery AND (Occlusion OR embolism))) OR "RAO" OR "CRAO" OR "BRAO" OR ((Retinal OR Ocular) AND Stroke))
3.	1 and 2

Table S4. Search strategy for Web of Science.

Original search: April 5, 2025, n = 185. Updated search: August 18, 2026, n = 196.

1.	TS=("Atrial Fibrillation" OR "AF" OR "cardiac arrhythmia" OR "irregular heartbeat" OR "heart rhythm disorder")
2.	TS=(("Retinal Artery Occlusion" OR "RAO" OR "CRAO" OR "BRAO" OR "retinal artery embolism") OR ("Retinal Stroke" OR "Ocular Stroke"))
3.	1 and 2

Table S5. Characteristics of studies reporting the prevalence of atrial fibrillation in retinal artery occlusion

Study	Year	Country	Study Design	Centers	Study setting	Type of RAO	Sample size	AF	Age (mean, years)	Male (%)	Method of diagnosis of AF	Patient inclusion period
**Hankey [29]**	1991	United Kingdom	Prospective cohort	Single	Hospital-based	RAO	98	4	64	56	ECG-confirmed	1977-1986
**Mead [30]**	2002	United Kingdom	Cross-sectional	Single	Hospital-based	RAO	79	3	65	59	ECG-confirmed	1994-1999
**Rudkin a [32]**	2010	United States and Australia	Case series	Multi	Hospital-based	CRAO	40	3	56.6 (JHH cohort), 75.9 (FMC cohort)	57.5	medical history	1996-2006
**Rudkin b [31]**	2010	Australia	Case series	Single	Hospital-based	CRAO	33	3	73	NS	medical history	1997-2008
**Coisy [33]**	2013	France	Case series	Multi	Hospital-based	CRAO, BRAO, RAO	131	19	69.5	64.1	ECG-confirmed	2001-2012
**Fieß [35]**	2014	Germany	Case series	Single	Hospital-based	CRAO	74	6	73.9	52.7	medical history	2000-2012
**Leisser [34]**	2014	Austria	Cross-sectional	Single	Hospital-based	RAO	110	12	72*	63.6	medical history	2006-2013
**Callizo [37]**	2015	Germany	Cross-sectional	Multi	Hospital-based	CRAO	77	15	62.2	70	ECG-systematic	2002-2007
**Chang [36]**	2015	Taiwan	Retrospective cohort	Multi	Registry-based	RAO	688	8	61.3	54.8	ICD-9	1999-2008
**Chapin [38]**	2015	United States	Cross-sectional	Multi	Hospital-based	RAO	17	0	51	41.2	Medical records	2001-2011
**Rim [39]**	2016	South Korea	Retrospective cohort	Multi	Registry-based	RAO	401	36	NS	54.6	KCD	2004-2013
**Callizo [28]**	2017	Germany	Cross-sectional	Single	Hospital-based	RAO	25	4	68.1	68	ECG, 7-day Holter	2011-2012
**French [41]**	2018	United States	Retrospective cohort	Multi	Registry-based	CRAO	3338	713	NS	52.3	ICD-9	2013
**Lavin [21]**	2018	United States	Retrospective cohort	Single	Hospital-based	CRAO	103	14	65.1	54.4	telemetry, selective 30-day monitoring	2009-2017
**Schultheiss [40]**	2018	Germany	Case series	Single	Hospital-based	CRAO	20	1	72.8	50	ECG (systematic 24h monitoring)	2014-2016
**Avery [43]**	2019	Canada	Case series	Single	Hospital-based	CRAO, BRAO	31	3	74.7	64.5	Medical records	2008-2013
**Mir [44]**	2019	United States	Cross-sectional	Multi	Registry-based	CRAO	17117	2689	68.2	47	ICD-9	2013-2014
**Kang [42]**	2019	Taiwan	Retrospective cohort	Multi	Registry-based	CRAO	3778	36	62.7 (aspirin), 61.9 (aspirin-naive)	59.05	ICD-9	1998-2013
**Danyel [52]**	2020	Germany	Case-control	Single	Hospital-based	CRAO	20	2	60.6	55	Medical records	2015-2019
**Laczynski [46]**	2020	United States	Retrospective cohort	Single	Hospital-based	RAO	221	4	66.1	52.9	Medical records	2004-2018
**Mac Grory [47]**	2020	United States	Prospective cohort	Multi	Hospital-based	CRAO	112	14	65.5	53.6	ECG, 24h telemetry; selective extended monitoring	2009-2019

Study	Year	Country	Study Design	Centers	Study setting	Type of RAO	Sample size	AF	Age (mean, years)	Male (%)	Method of diagnosis of AF	Patient inclusion period
**Shaikh [48]**	2020	United States	Cross-sectional	Multi	Registry-based	RAO	19809	2543	NS	55	ICD-9	2002-2013
**Schorr [45]**	2020	United States	Cross-sectional	Multi	Registry-based	RAO	4871	761	66.8	51.3	ICD-9	2013-2015
**Hoyer [49]**	2021	Germany	Case series	Single	Hospital-based	CRAO	101	22	72.13	57.6	Medical records	2016-2020
**Ruiz-Ares [50]**	2021	Spain	Cross-sectional	Single	Hospital-based	CRAO	24	3	69.2	66.7	ECG-systematic	2011-2018
**Scoles [55]**	2021	United States	Retrospective cohort	Multi	Registry-based	CRAO, BRAO, RAO	16193	3110	74.6	49.4	ICD-9; ICD-10	2004-2016
**Vestergaard [53]**	2021	Denmark	Retrospective cohort	Multi	Registry-based	RAO	6628	699	69.8	54.6	ICD-8; ICD-10	2000-2018
**Uppuluri [51]**	2021	United States	Case-control	Multi	Registry-based	RAO	522	5	37.3	56.6	ICD	2002-2014
**Chodnicki [54]**	2022	United States	Case series	Single	Population-based	CRAO	89	9	74.6	56.2	Medical records	1976-2016
**Feltgen [61]**	2022	Germany	Case-control	Multi	Hospital-based	CRAO, BRAO	56	10	74.5	41.1	Medical records	2021
**Jurado [57]**	2022	Switzerland	Cross-sectional	Multi	Registry-based	CRAO	397	51	71.8	62.7	medical history	2014-2019
**Lai [63]**	2022	Taiwan	Case–control	Single	Hospital-based	RAO	82	8	61.5	70	Medical records	2011-2020
**Ørskov a clinical [59]**	2022	Denmark	Case–control	Multi	Registry-based	RAO	5312	428	68.4	55.6	ICD-10	2000-2018
**Ørskov b evaluation [62]**	2022	Denmark	Retrospective cohort	Multi	Registry-based	RAO	7906	713	69.2	53.1	ICD-10	1995-2018
**Raber [65]**	2022	Germany	Case series	Single	Hospital-based	CRAO	37	6	68.7 (IVT group), 71.8 (CTG)	65	24-h ECG systematic	2010-2020
**Schönecker [60]**	2022	Germany	Case series	Single	Hospital-based	RAO	16	2	72.4	56.2	telemetry; selective extended monitoring	2015-2020
**Suri [64]**	2022	United States	Retrospective cohort	Multi	Registry-based	RAO	14527	1945	68.7	52	ICD-10	2016-2018
**Yousuf [56]**	2022	United States	Cross-sectional	Multi	Registry-based	RAO	2802	260	NS	53.5	ICD-9	2006-2014
**Roskal-Wałek [58]**	2022	Poland	Case-control	Single	Hospital-based	RAO	139	21	70	63.3	Medical records	2004-2020
**Balla [69]**	2023	Hungary	Retrospective cohort	Single	Hospital-based	CRAO, BRAO, RAO	169	32	64.1	57.4	Medical records	2009-2019
**Baumgartner [72]**	2023	Multinational (Europe)	Retrospective cohort	Multi	Hospital-based	RAO	81	8	65 (IVT group), 71 (non-IVT group)	56.8	Medical records	NS
**Hamedani [71]**	2023	United States	Cross-sectional	Multi	Registry-based	RAO	19773	2293	NS	54	ICD-9; ICD-10	2011-2018

Study	Year	Country	Study Design	Centers	Study setting	Type of RAO	Sample size	AF	Age (mean, years)	Male (%)	Method of diagnosis of AF	Patient inclusion period
**Lange [73]**	2023	Germany and France	Cross-sectional	Multi	Hospital-based	CRAO	68	9	68.4	63.2	Medical records	2010-2020
**Roskal-Wałek [66]**	2023	Poland	Retrospective cohort	Single	Hospital-based	RAO	198	29	70.3	62.1	Medical records	2018-2023
**Smith [67]**	2023	Canada	Case series	Single	Hospital-based	CRAO	27	5	73.3	44.4	Medical records	2018-2020
**Smith and Kukolja [74]**	2023	Germany	Retrospective cohort	Single	Hospital-based	RAO	54	5	71.3	50	Medical records	2016-2020
**Williamson [70]**	2023	Australia	Case series	Single	Hospital-based	CRAO, BRAO	22	2	67.4	65	Medical records	2015-2021
**Yoo [68]**	2023	South Korea	Retrospective cohort	Multi	Registry-based	CRAO, BRAO, RAO	13843	276	60.1	57.1	ICD-10	2008-2020
**Alhayek [75]**	2024	United States	Retrospective cohort	Multi	Hospital-based	CRAO	20	2	68.5 (IVT group), 55.2 (IAT group)	70	Medical records	1997-2022
**Bustamante [77]**	2024	Spain	Prospective cohort	Single	Hospital-based	CRAO	49	14	75	51	ECG, 24h monitoring, extended monitoring when indicated	2018-2023
**Diel [76]**	2024	Germany	Cross-sectional	Single	Hospital-based	RAO	76	16	71.9	57.9	Medical records	NS
**Gilbert [79]**	2024	United States	Case series	Multi	Hospital-based	CRAO	86	16	72	60.5	Medical records	2021-2023
**Subah [78]**	2024	United States	Cross-sectional	Multi	Registry-based	CRAO	5009	991	68.6	51.8	ICD-9; ICD-10	2010-2019
**Akosman [81]**	2025	United States	Cross-sectional	Multi	Registry-based	CRAO	3736	560	69	53	ICD-10	2016-2021
**Liu [80]**	2025	China	Prospective cohort	Single	Hospital-based	RAO	335	35	NS	57.3	Medical records	2019-2023
**Chen [83]**	2026	United States	Retrospective cohort	Single	Hospital-based	RAO	56	8	64*	60.7	Medical records	2014-2024
**Huemer [84]**	2026	United Kingdom	Retrospective cohort	Single	Hospital-based	CRAO, BRAO	232	32	NS	NS	ICD-10	2011-2018
**Ryan [82]**	2026	Multinational (Europe)	RCT	Multi	Hospital-based	CRAO	78	2	71 (tenecteplase group), 72 (aspirin group)	65.4	Medical records	2020-2025

*Median, AF = atrial fibrillation; BRAO = branch retinal artery occlusion; CRAO = central retinal artery occlusion; CTG = conservative treatment group; ECG = electrocardiography; FMC = Flinders Medical Centre cohort; IAT = intra-arterial thrombolysis; ICD = International Classification of Diseases; IVT = intravenous thrombolysis; JHH = Johns Hopkins Hospital cohort; NS = not specified; RAO = retinal artery occlusion; RCT = Randomized Controlled Trial

Table S6. Characteristics of studies reporting incident atrial fibrillation after retinal artery occlusion

Author	Year	Country	Study Design	Centers	Study setting	Type of RAO	Sample size	Incident AF	Age	Male (%)	Method of diagnosis of AF	AF definition	Follow-up duration	Study date
**Christiansen [17]**	2018	Denmark	Retrospective cohort	Multi	Registry-based	RAO	706	74	70.7 (61.2–78.7)	49.7	ICD-8; ICD-10	NS	until AF, death, or end of study	1 January 1997-31 December 2011
**Watson [87]**	2020	United States	Retrospective cohort	Single	Hospital-based	RAO	46	7	65.1 ± 13.7	45.7	Implantable loop recorder	Episode of irregular rhythm without P waves detected by ILR (≥2 min)	43.2 months	January 2012-March 2019
**Mac Grory [85]**	2021	United States	Retrospective cohort	Multi	Linked EHR database and device registry	CRAO	100	41	69.0 ± 14.0	57	Implantable cardiac monitoring (ICM, IPG, ICD, CRT-P, CRT-D)	AF episode ≥2 min detected by implantable cardiac device	24 months	January 2007-June 2019
**Vonderlin [86]**	2021	Germany	Retrospective cohort	Single	Hospital-based	RAO	174	22	74.2 ± 13.0	61.5	ECG or ≥24-h Holter	AF documented by ECG or Holter	20 ± 12 months	January 2014-November 2016
**Huemer [84]**	2026	United Kingdom	Retrospective cohort	Single	Hospital-based	CRAO/ BRAO	136/ 96	19/ 7	NS	NS	ICD-10	NS	NS	1 August 2011-31 March 2018

RAO = retinal artery occlusion; AF = atrial fibrillation; ICD-8 = International Classification of Diseases, 8th revision; ICD-10 = International Classification of Diseases, 10th revision; NS= not specified; ILR = implantable loop recorder; CRAO = central retinal artery occlusion; EHR = electronic health record; ICM = insertable cardiac monitor; IPG =implantable pacemaker generator; ICD = implantable cardioverter-defibrillator; CRT-P = cardiac resynchronization therapy device-pacemaker; CRT-D = cardiac resynchronization therapy device-defibrillator.

Figure S1. Traffic light plot of risk of bias using the Joanna Briggs Institute checklist for prevalence studies (cohort and cross-sectional studies)

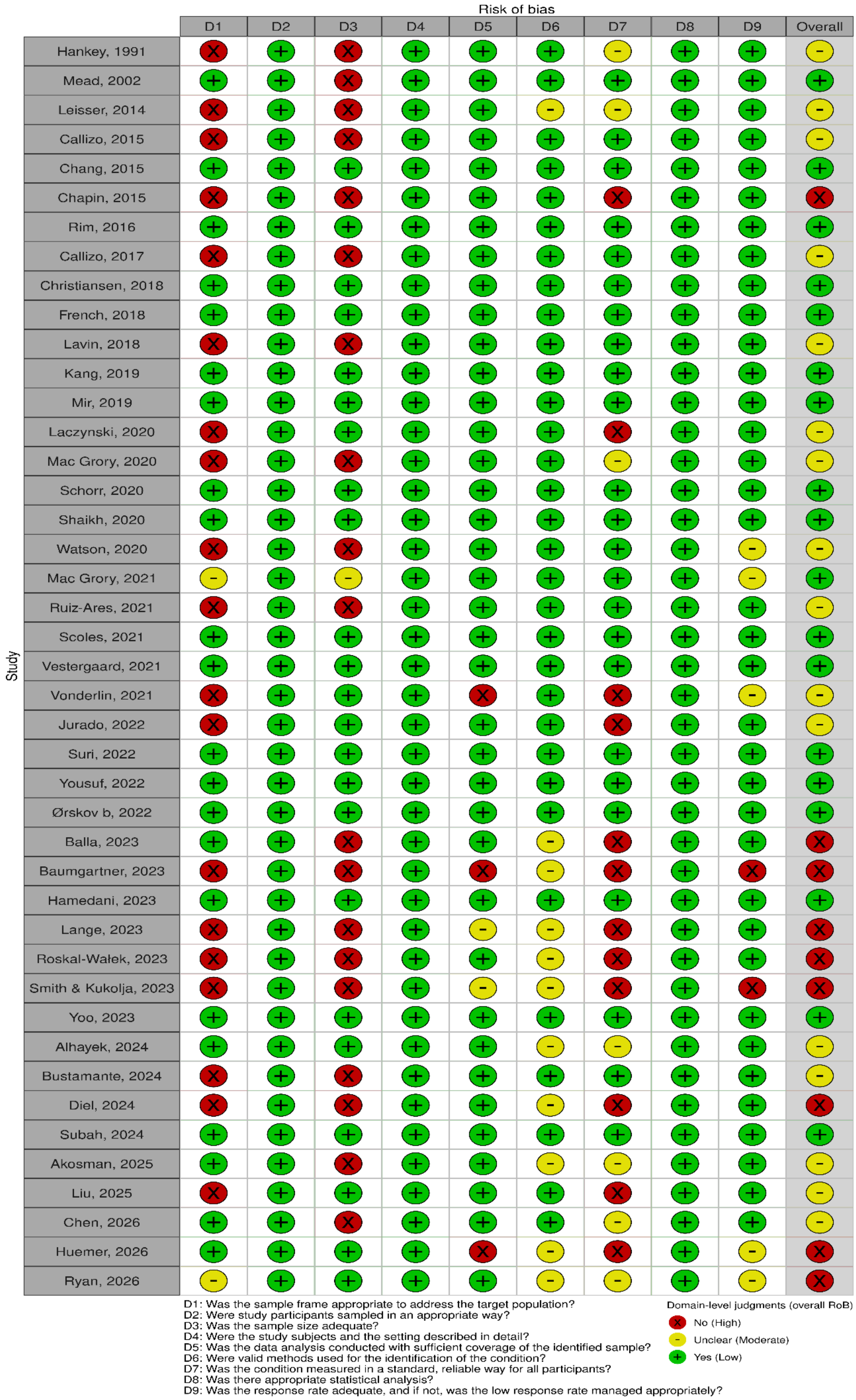

Figure S2. Traffic light plot of risk of bias using the Joanna Briggs Institute checklist for case series

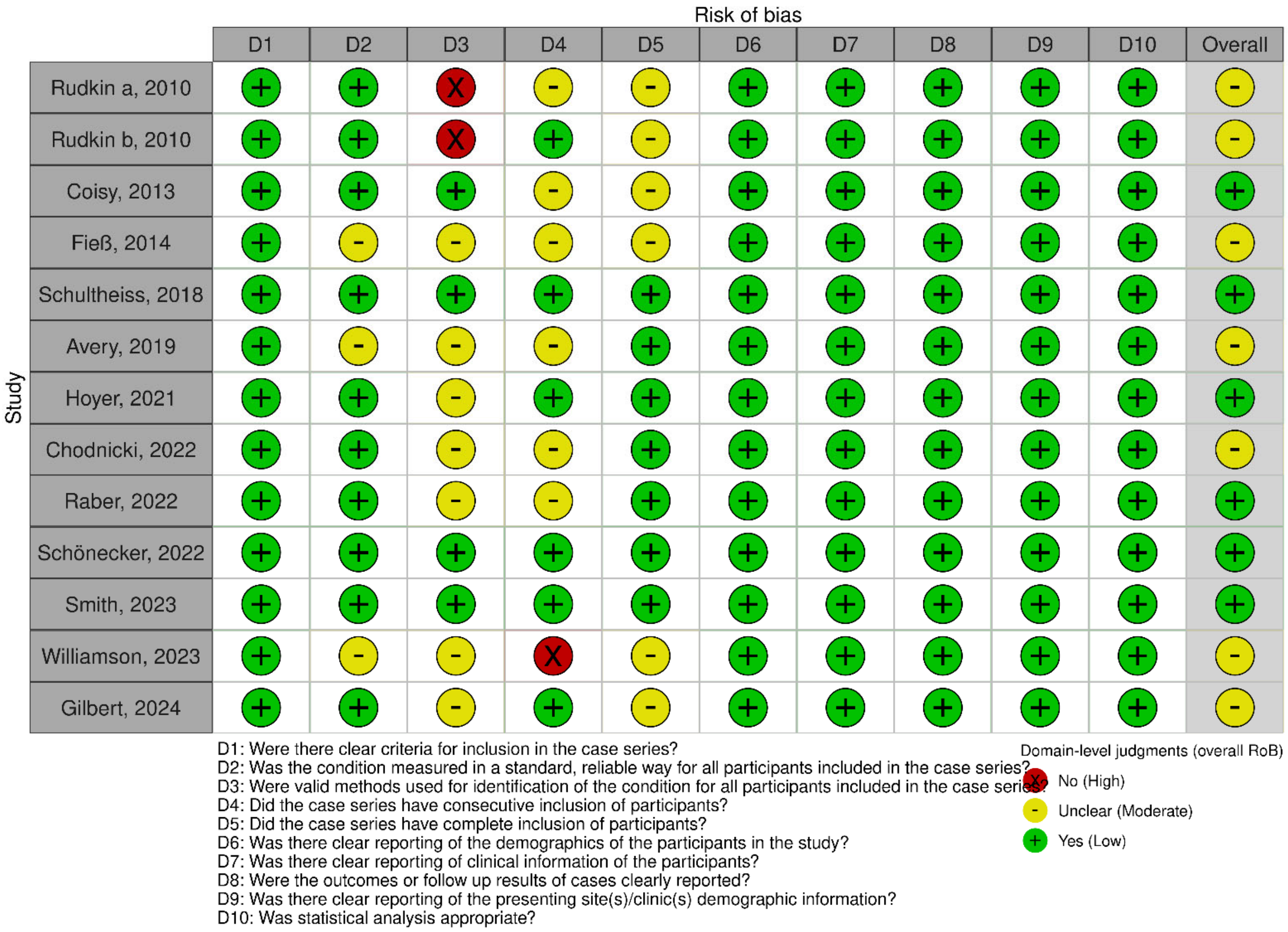

Figure S3. Traffic light plot of risk of bias using the Joanna Briggs Institute checklist for case-control studies

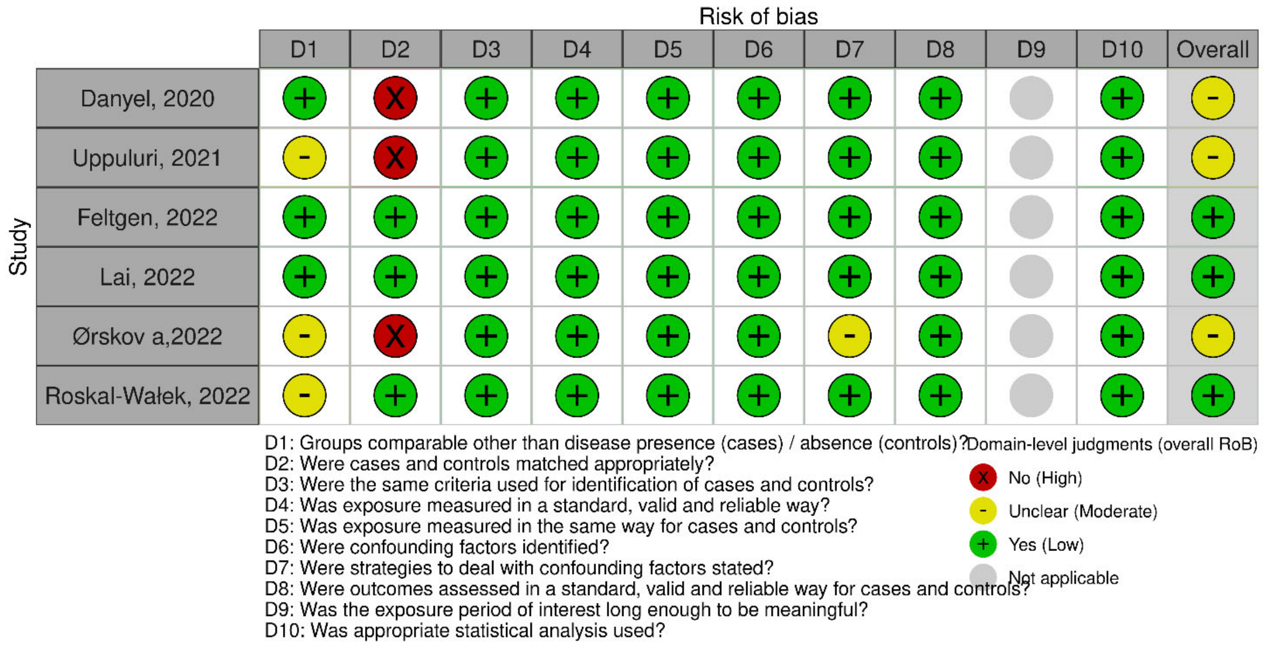

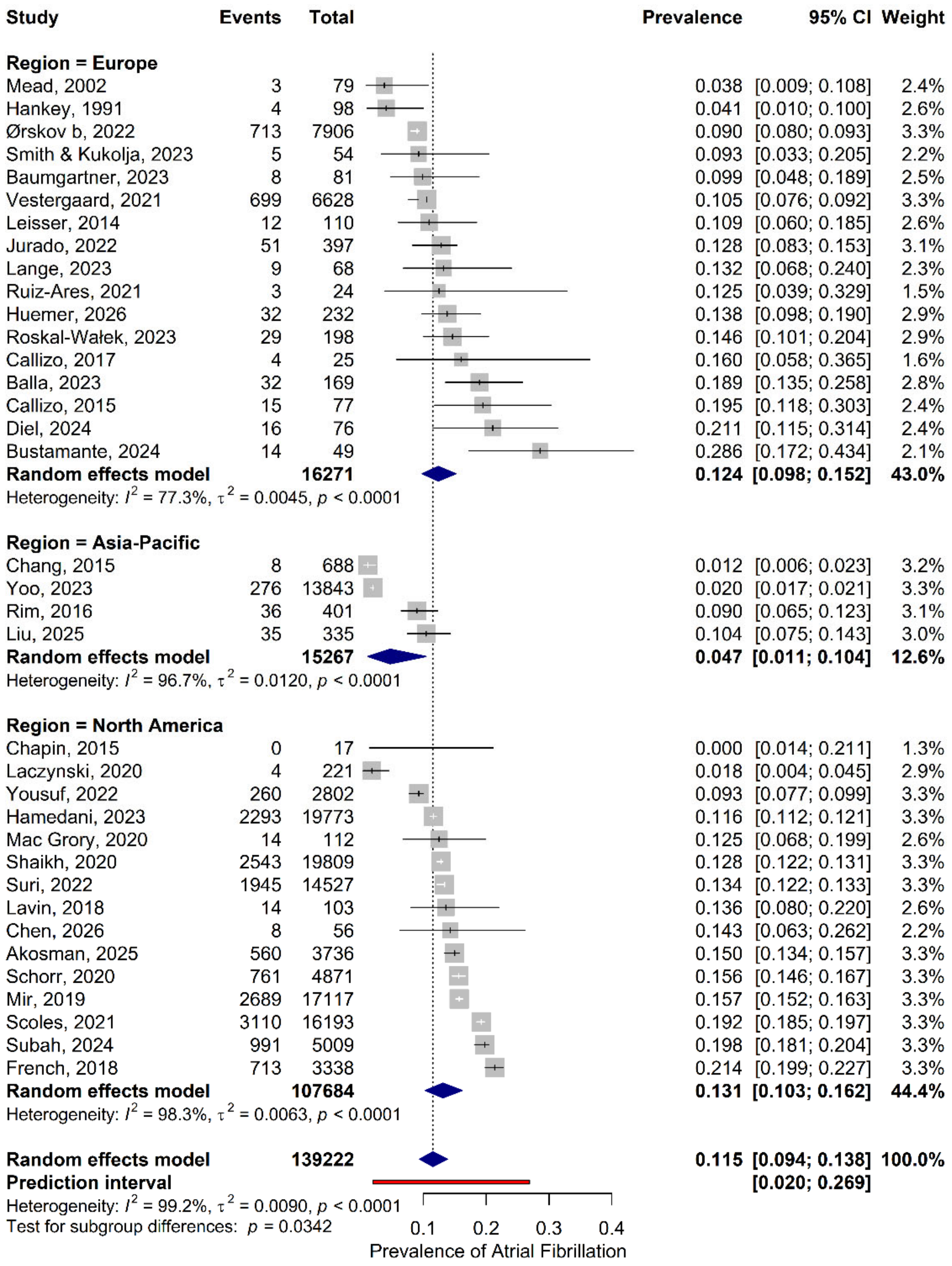

Figure S4. Prevalence of atrial fibrillation in retinal artery occlusion by region

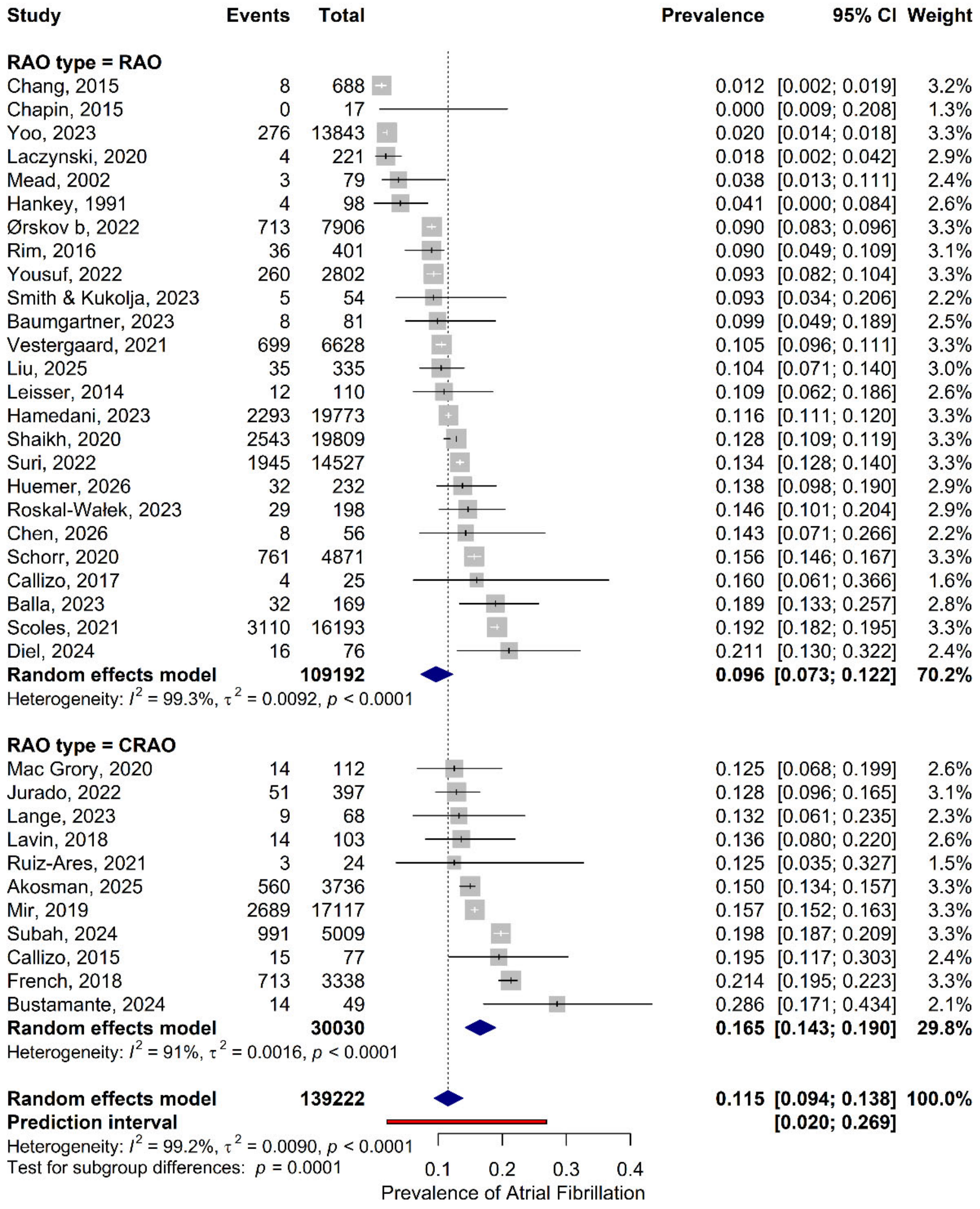

Figure S5. Prevalence of atrial fibrillation in retinal artery occlusion type of retinal artery occlusion.

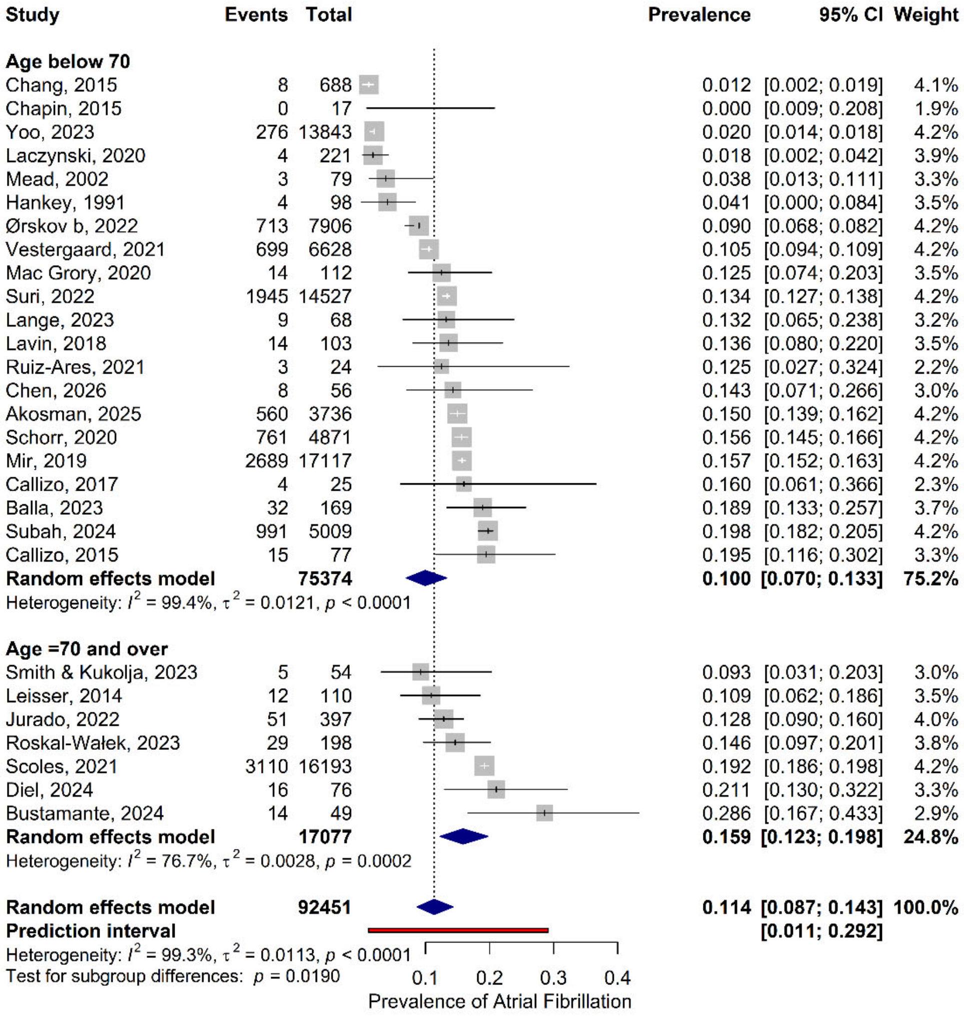

Figure S6. Prevalence of atrial fibrillation in retinal artery occlusion by mean age of study participants.

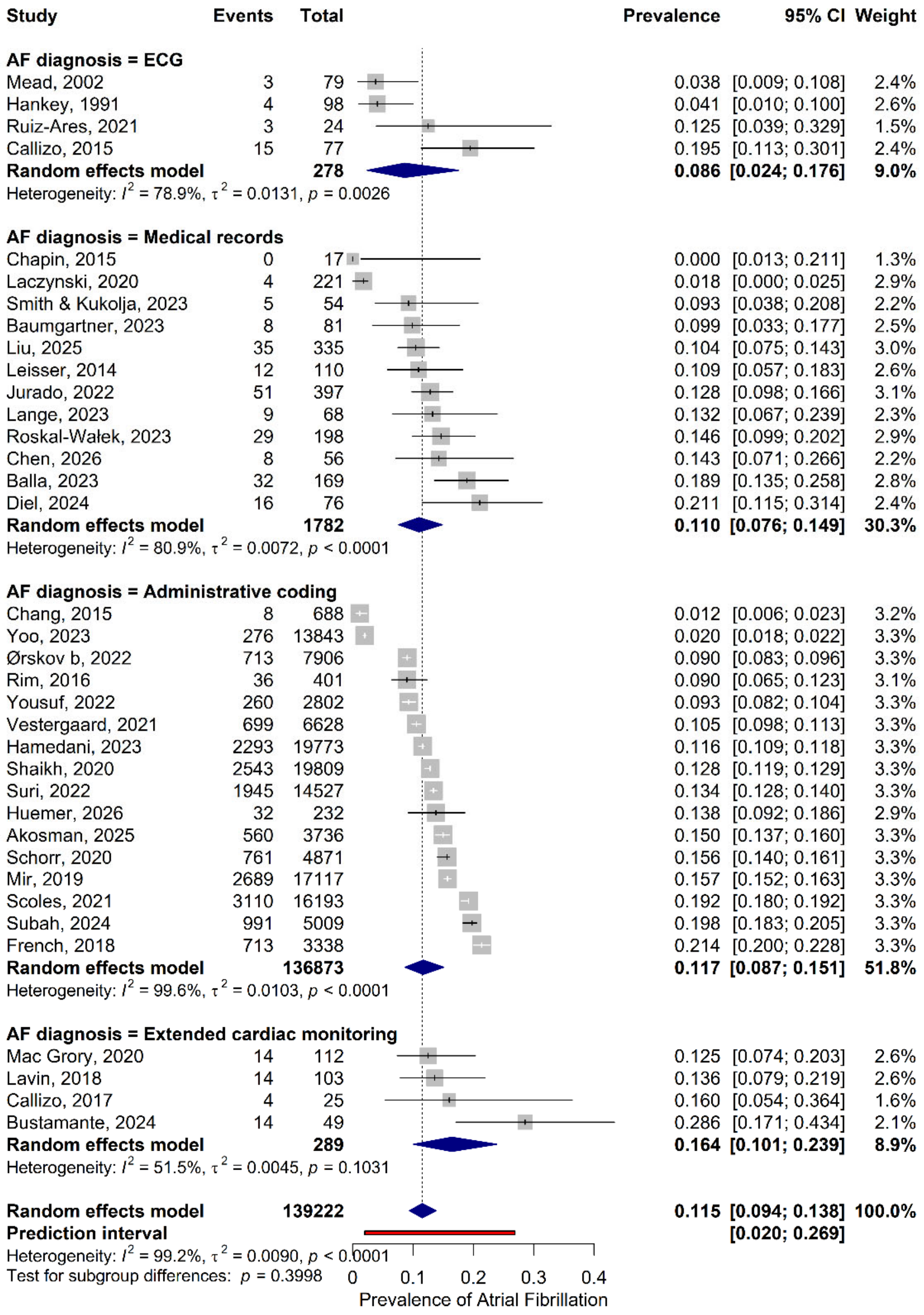

Figure S7. Prevalence of atrial fibrillation in retinal artery occlusion by method of diagnosis of atrial fibrillation.

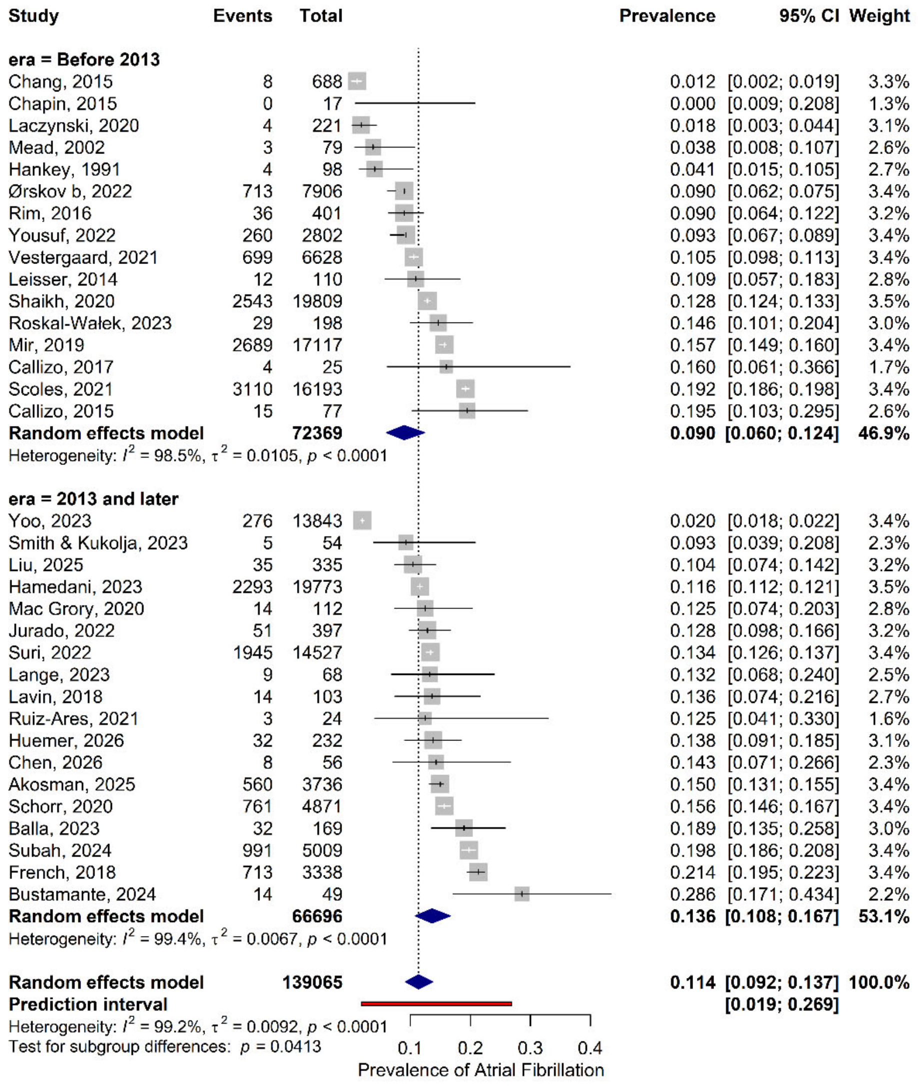

Figure S8. Prevalence of atrial fibrillation in retinal artery occlusion by study period.

** Stratified by study-period midpoint (mean of enrolment start and end years), dichotomised at 2013, coinciding with the AHA/ASA statement classifying RAO as a stroke-equivalent condition

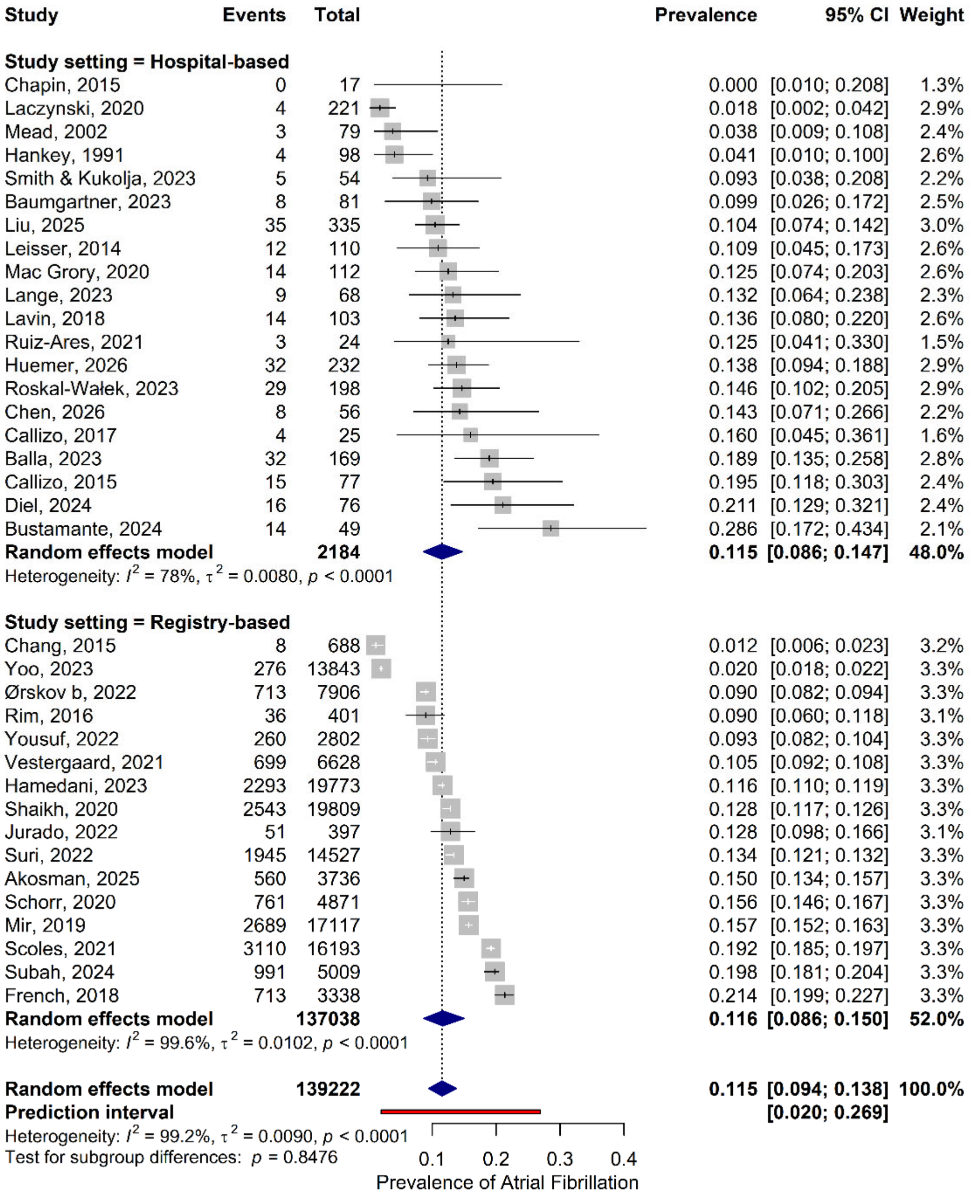

Figure S9. Prevalence of atrial fibrillation in retinal artery occlusion by study setting.

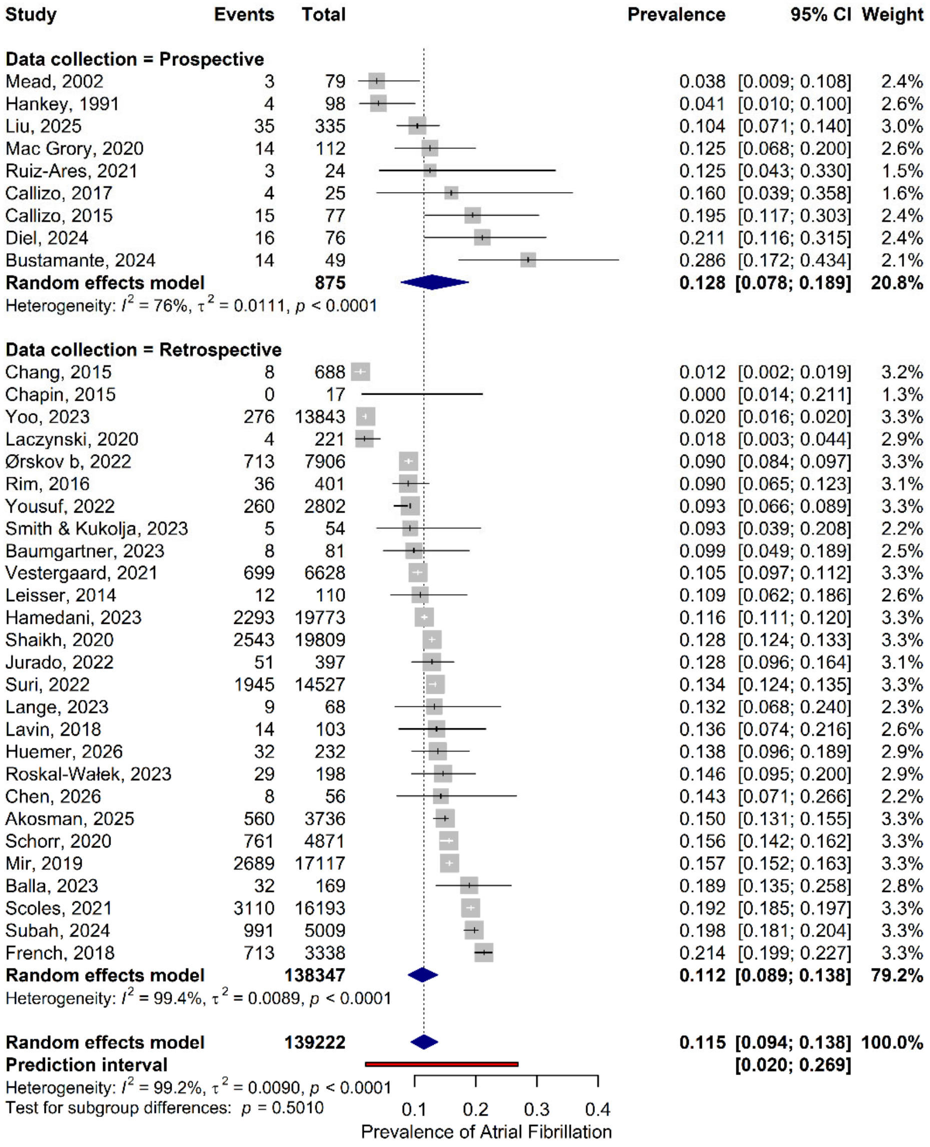

Figure S10. Prevalence of atrial fibrillation in retinal artery occlusion by timing of data collection.

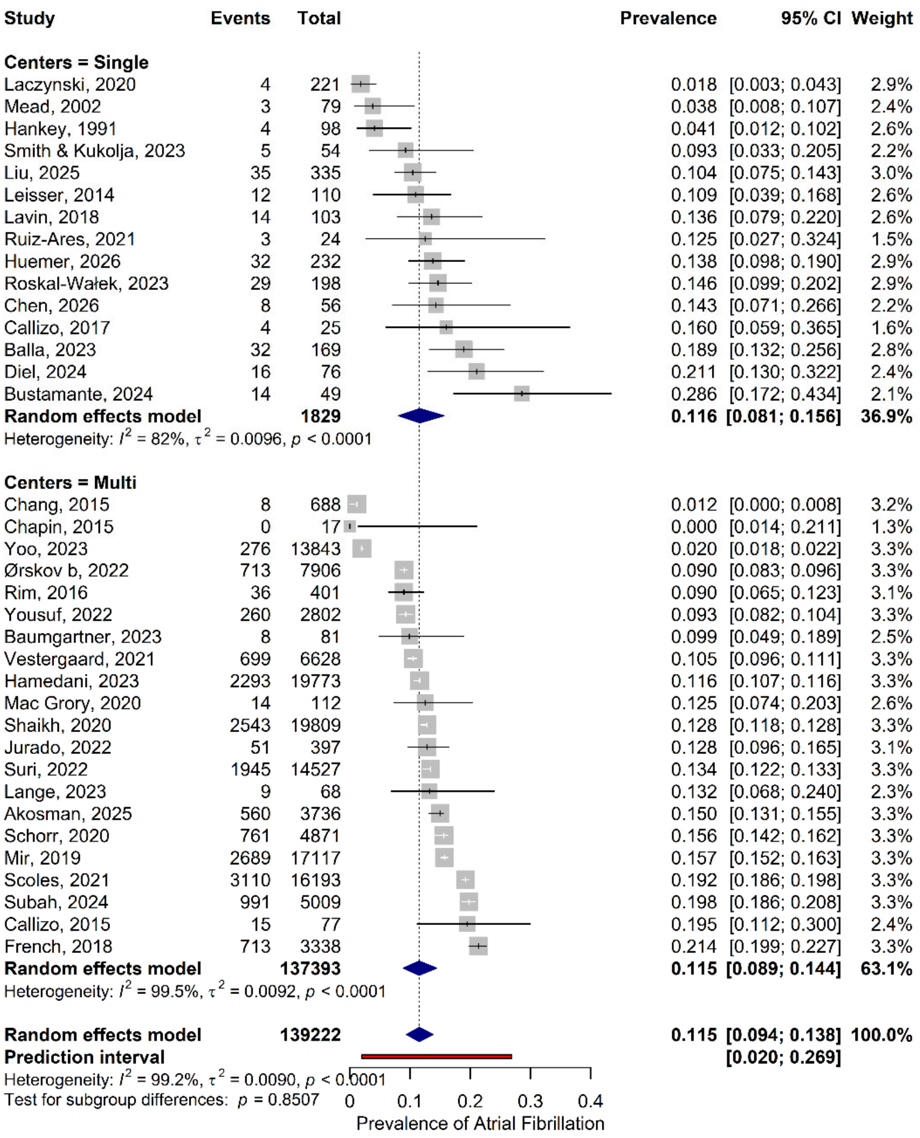

Figure S11. Prevalence of atrial fibrillation in retinal artery occlusion by number of centers.

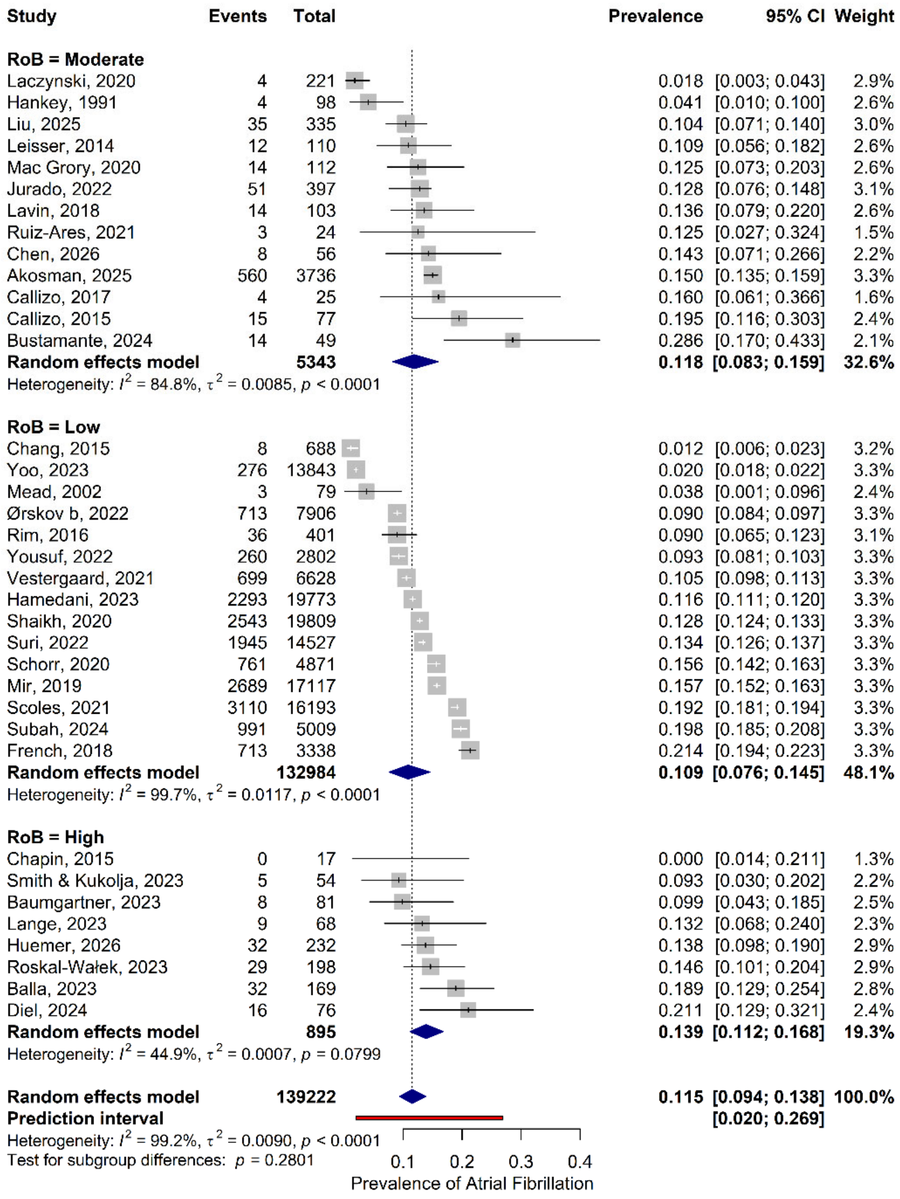

Figure S12. Prevalence of atrial fibrillation in retinal artery occlusion by risk of bias in individual studies

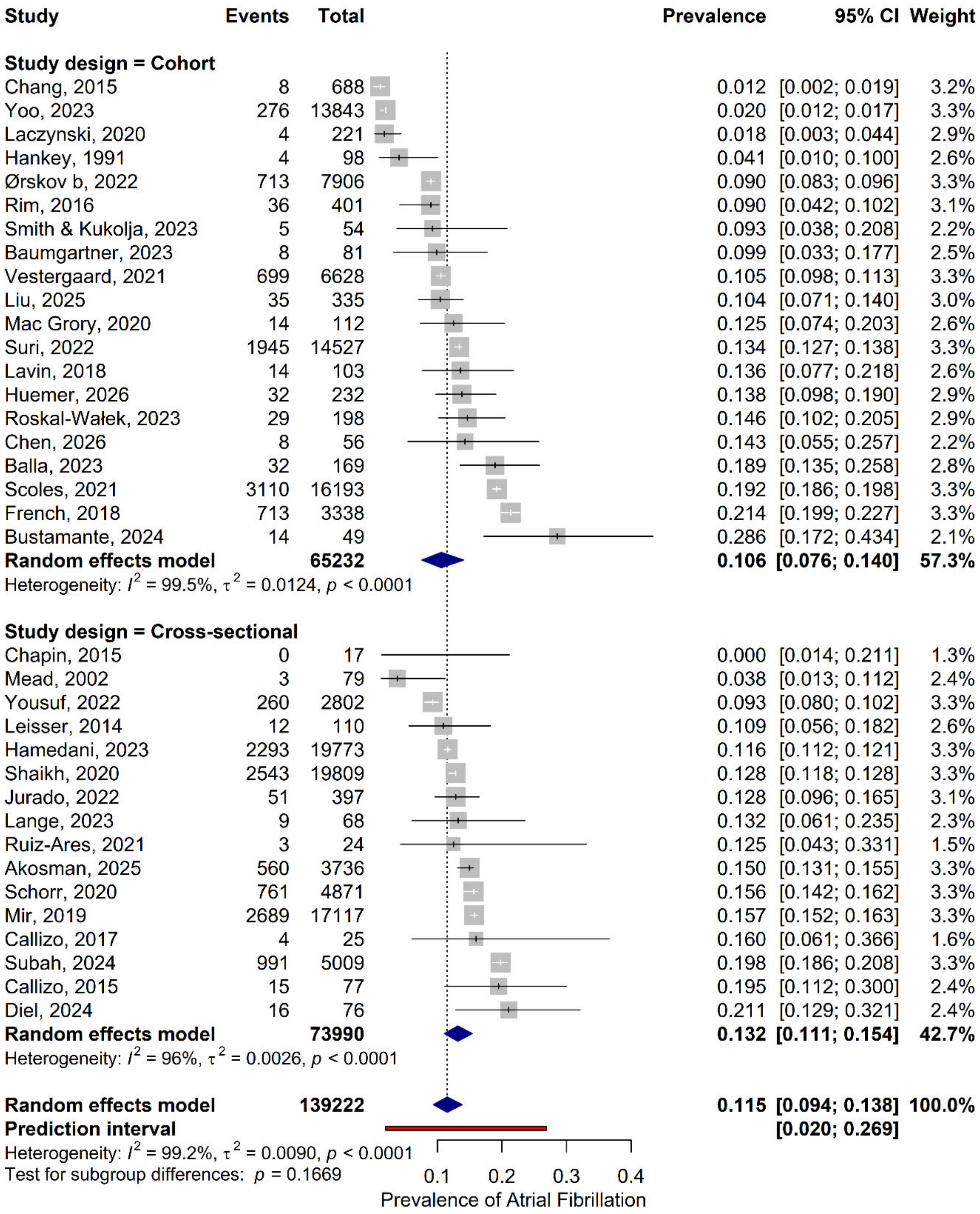

Figure S13. Prevalence of atrial fibrillation in retinal artery occlusion by study design.

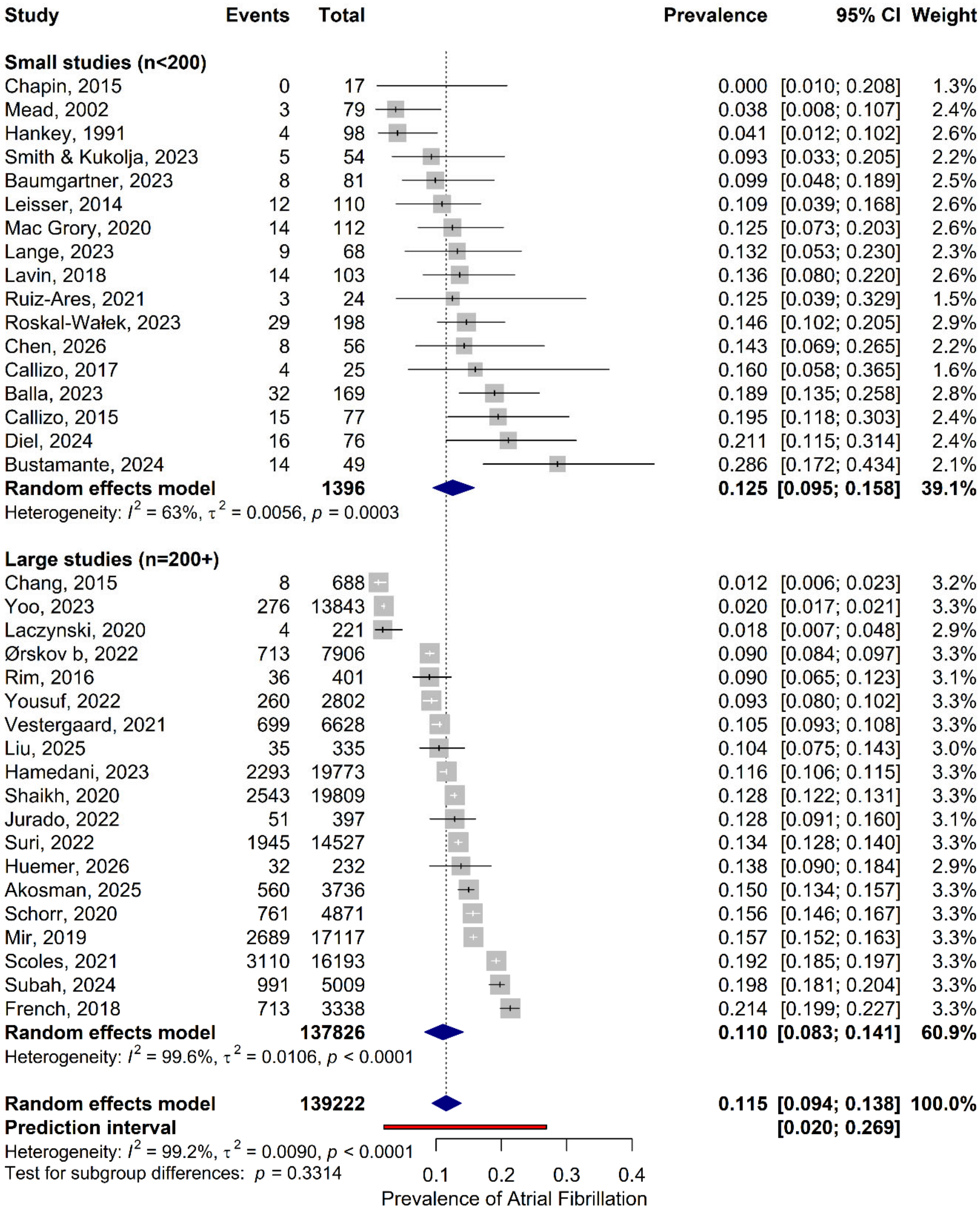

Figure S14. Prevalence of atrial fibrillation in retinal artery occlusion by sample size

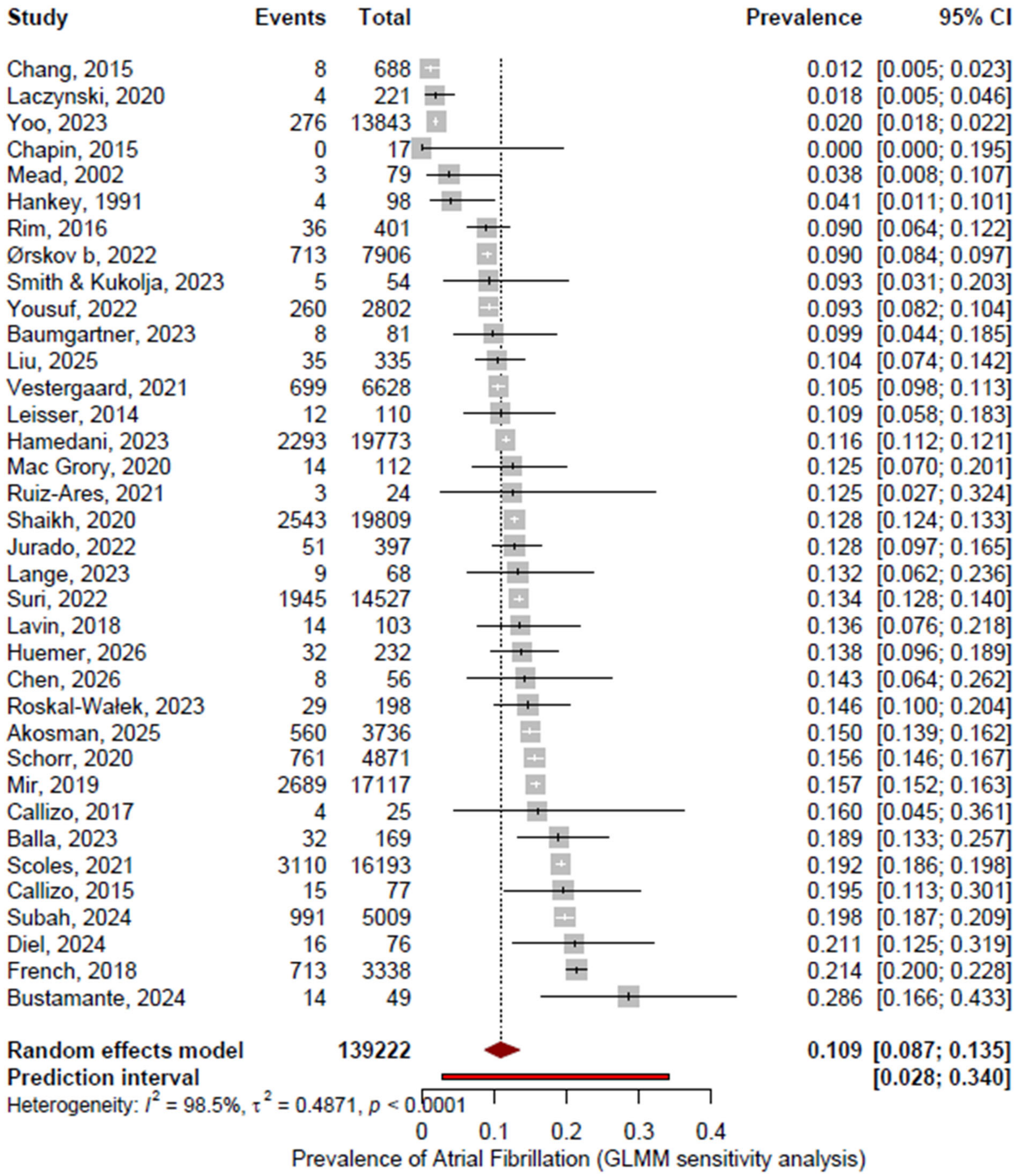

Figure S15: Sensitivity analysis re-estimating the pooled prevalence of atrial fibrillation using a generalised linear mixed model.

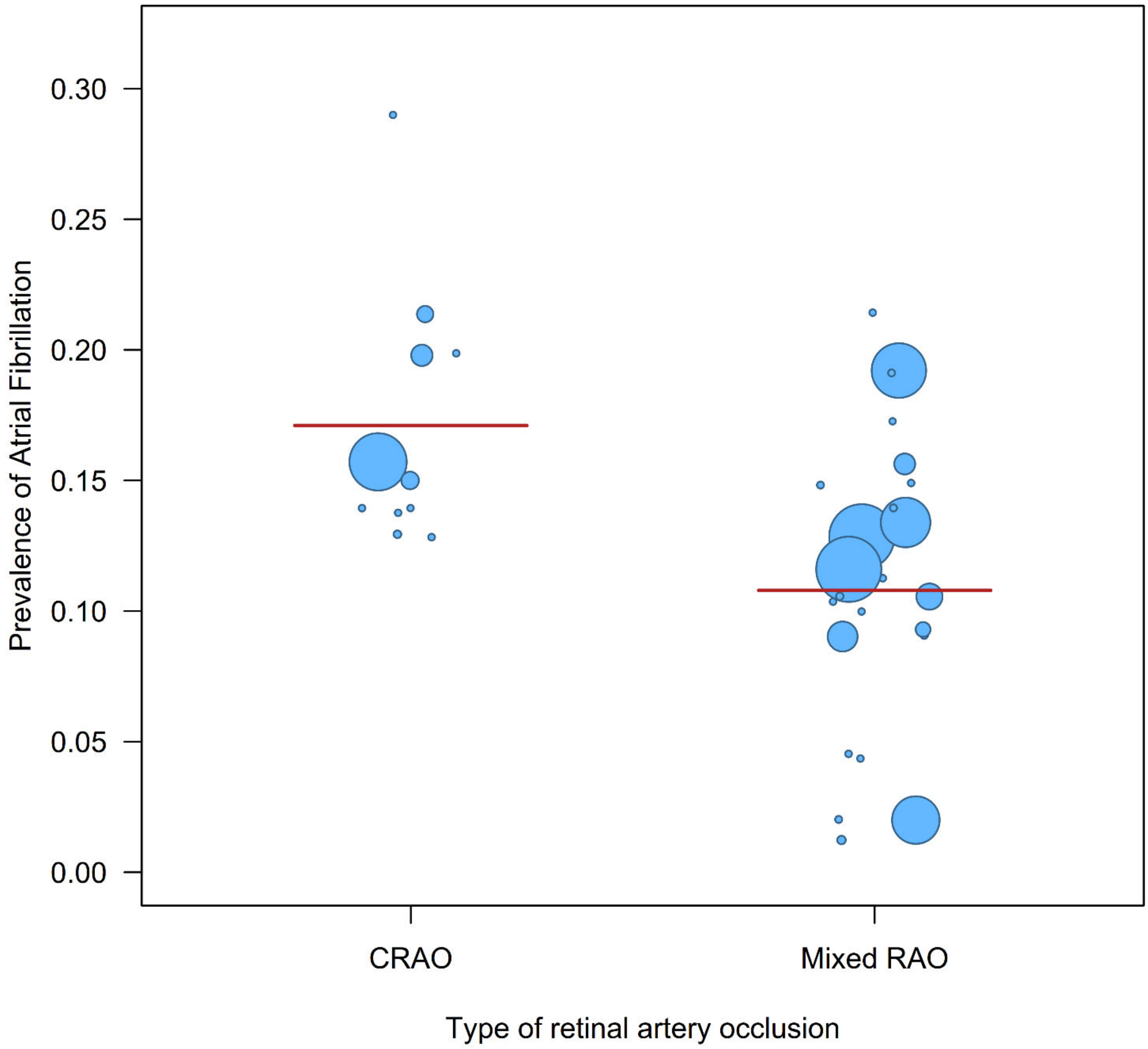

Figure S16. Bubble plot of atrial fibrillation prevalence by type of retinal artery occlusion.

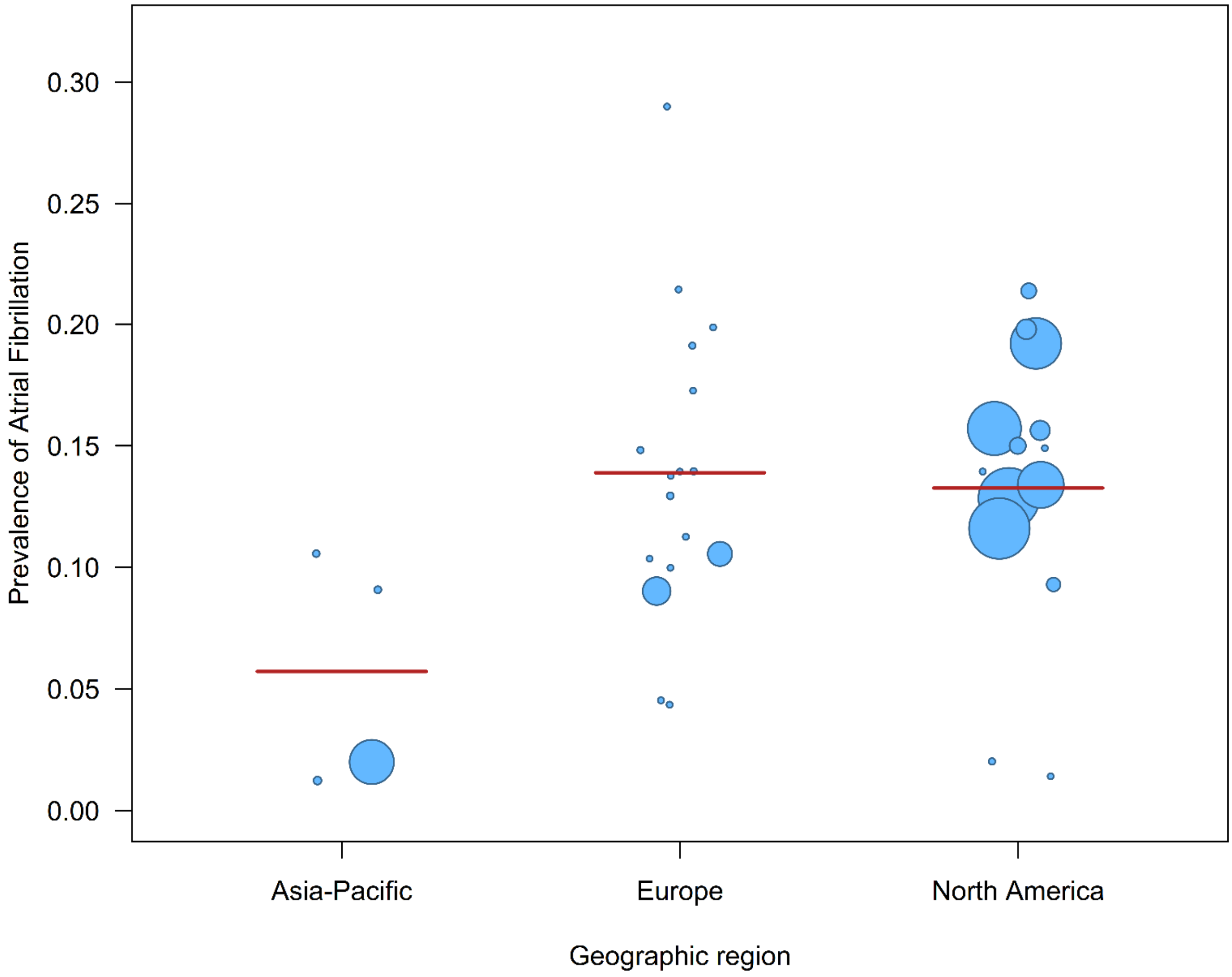

Figure S17. Bubble plot of atrial fibrillation prevalence by geographic region.

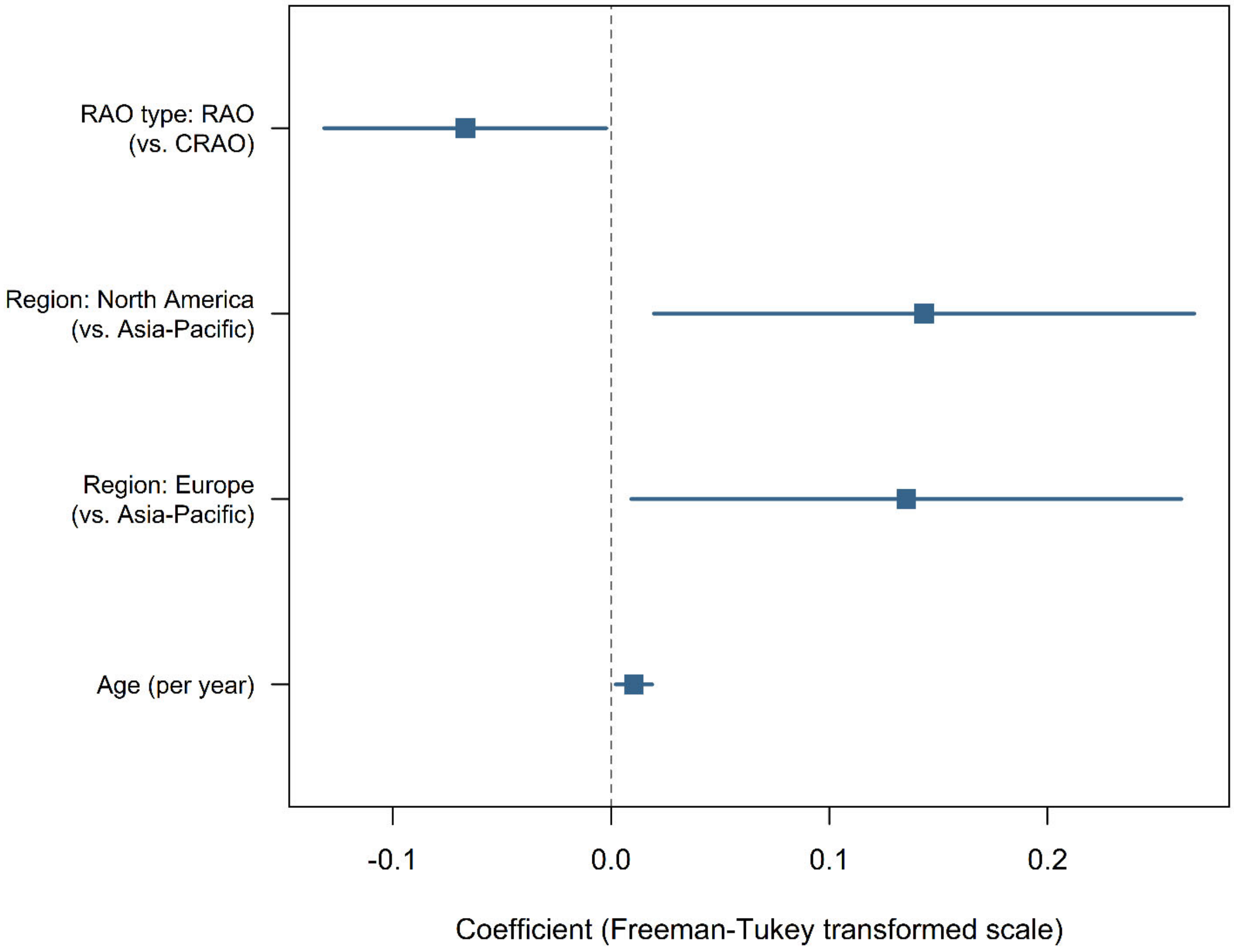

Figure S18. Forest plot of coefficients from the multivariable meta-regression model..

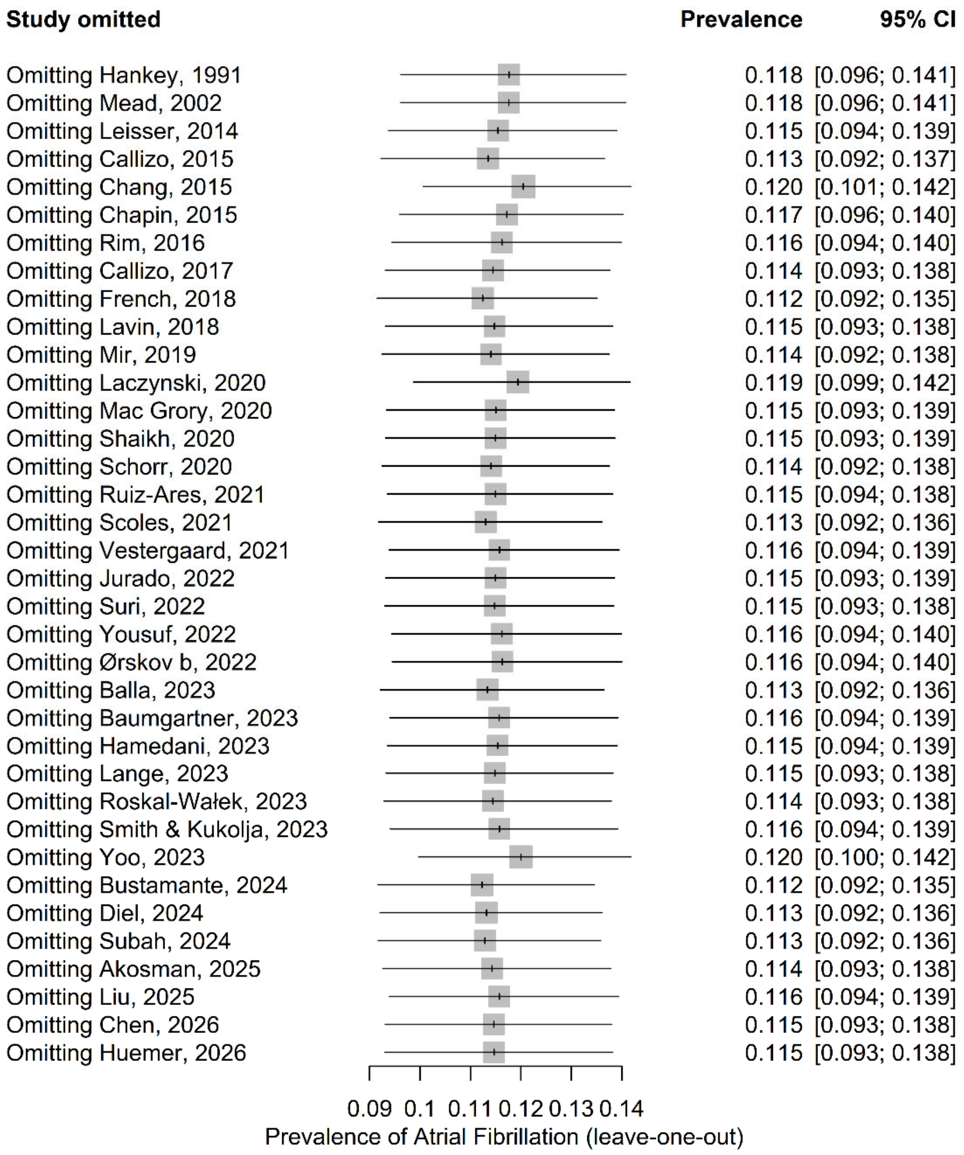

Figure S19. Leave-one-out sensitivity analysis illustrating the influence of each individual study on the pooled prevalence estimate.

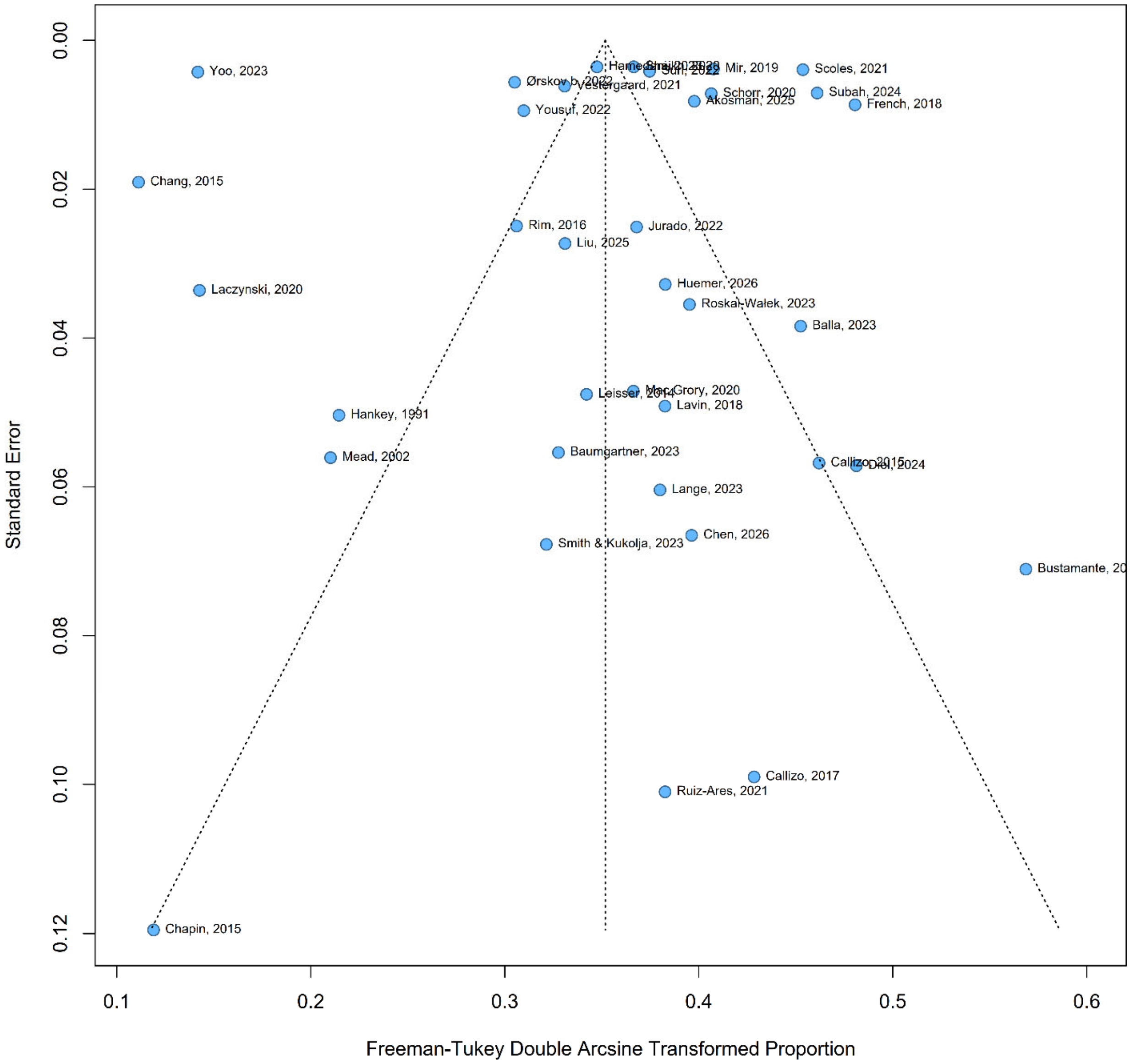

Figure S20. Funnel plot evaluating potential publication bias among studies included in the meta-analysis of atrial fibrillation prevalence in retinal artery occlusion

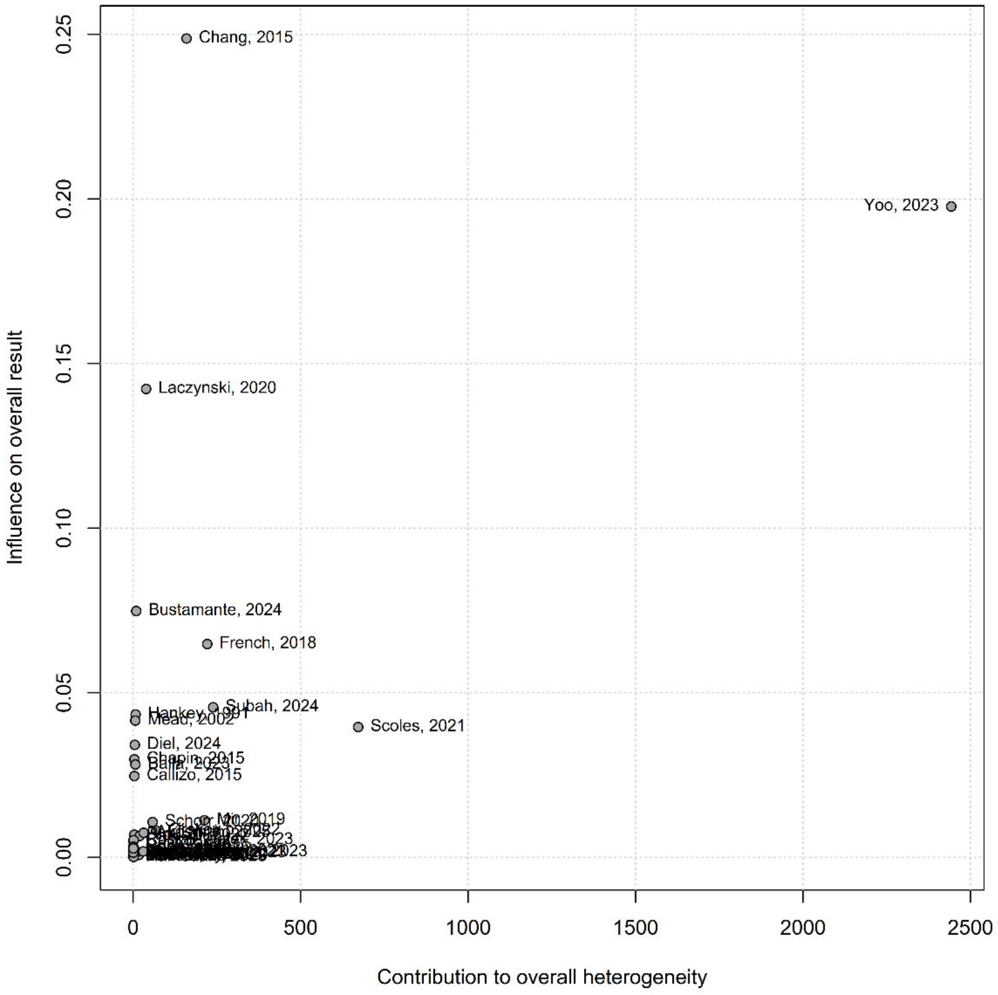

Figure S21. Baujat plot showing the contribution of individual studies to heterogeneity in the prevalence meta-analysis.

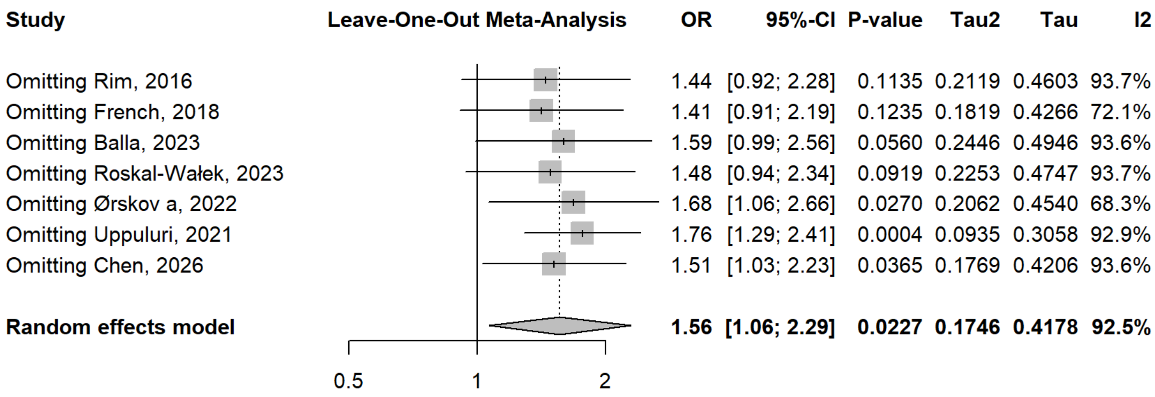

Figure S22. Leave-one-out sensitivity analysis of the pooled association between atrial fibrillation and retinal artery occlusion.

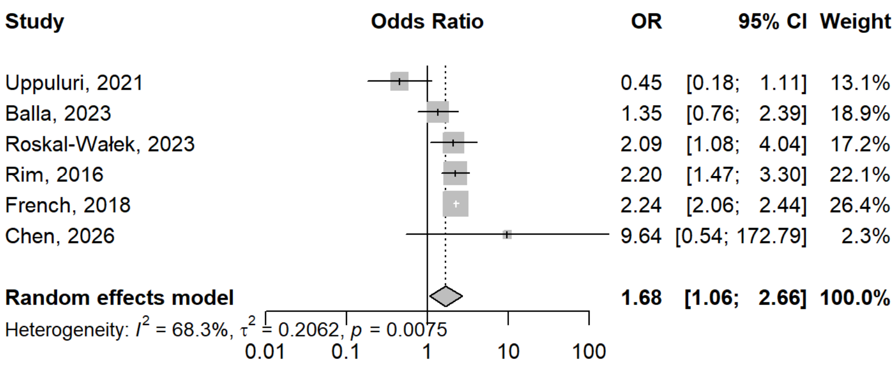

Figure S23. Sensitivity analysis restricted to studies reporting crude estimates of the association between atrial fibrillation and retinal artery occlusion.
